# Regenerative potential of immune cells after traumatic muscle injury

**DOI:** 10.3389/fimmu.2025.1610119

**Published:** 2025-09-17

**Authors:** Su Pu, Guangmin Hu, Yulu Cao, Guoming Shen, Yuqing Wang

**Affiliations:** School of Integrated Chinese and Western Medicine, Anhui University of Chinese Medicine, Hefei, China

**Keywords:** traumatic muscle injury, immune cell, neutrophil, macrophage, T lymphocytes

## Abstract

Traumatic muscle injury (TMI) causes significant morbidity and socioeconomic burden. Immune cells are central to the subsequent regenerative response, orchestrating dynamic interactions between innate and adaptive immunity. This review systematically summarizes the current understanding of the roles of key immune cells (neutrophils, macrophages, eosinophils, basophils, T lymphocytes, B lymphocytes) in TMI pathophysiology and repair, based on a comprehensive analysis of recent literature. Their intrinsic mechanisms, contributions to tissue regeneration, and therapeutic implications are discussed. Furthermore, we explore therapeutic strategies targeting immune cells, including biomaterials, pharmacologic interventions, cell therapies, and physical modalities. The aim of this review is to provide a consolidated understanding of immune-mediated repair mechanisms in TMI and to identify critical knowledge gaps and future research directions necessary for developing novel immunomodulatory therapies to optimize muscle regeneration and functional recovery.

## Introduction

Traumatic muscle injury comprises a range of skeletal muscle pathologies induced by direct trauma to muscle tissue, characterized by structural disruption and potential loss of function (1, 2). Such injuries have a significant impact on athletes (3), military personnel (4), and the elderly population (5), contributing not only to pain and functional limitations but also to a significant socio-economic burden. In the United States, the annual healthcare expenditure associated with sports-related injuries has been reported to reach up to 55.1 billion USD (6). TMI also reduces productivity and diminishes social participation, further compounding its economic impact (5, 6). Beyond the acute phase, traumatic muscle injuries frequently result in chronic complications such as fibrosis and muscle atrophy, which may predispose individuals to secondary osteoarthritis and significantly reduce long-term quality of life (7).

According to the international consensus classification system outlined in the Munich Declaration, these injuries are systematically categorized into four grades based on severity and pathological progression (8). As shown in **Table 1**, Grade I injuries involve minimal muscle fiber disruption (< 5%) without fascial rupture and are characterized by edema detectable on magnetic resonance imaging (MRI) during the acute inflammatory phase, accompanied by negligible functional impairment (8, 9, 16). Grade II injuries involve muscle fiber tears exceeding 5% and/or partial disruption of the fascia, accompanied by a moderate loss of function, transitioning from the acute to the subacute phase, requiring physical therapy and adjunctive interventions for recovery (11, 12, 17). Grade III injuries are characterized by complete muscle fiber rupture with retraction and a total loss of function, corresponding to the phase from subacute-to-regeneration phase and typically requiring surgical intervention (13, 18). Grade IV injuries involve complete rupture of both muscle fibers and the surrounding fascia, marking entry into the reconstruction phase and necessitating surgical structural repair (8, 14, 15).

**Table 1:** The grading system, pathological characteristics, and therapeutic interventions corresponding to the severity of TMI.

Grade	Muscle Fiber Damage	Fascial Tear	Inflammatory Phase	Functional Impairment	References
I	Mild (<5%)	None	Acute (MRI-visible edema)	Minimal	(8–10)
II	>5% or partial	Partial	Acute to subacute	Partial	(8, 11, 12)
III	Complete with muscle retraction	None	Subacute to regeneration	Total	(8, 13)
IV	Complete	Complete	Reconstruction	None	(8, 14, 15)

The interplay between TMI, therapeutic interventions, and the immune system is governed by an orchestrated network of immune cells and molecular mediators (1). This review focuses on the roles of immune cells in the repair and regeneration of traumatic muscle injuries. Specifically, the functions of each type of immune cell are outlined as follows. This search covered multiple databases, including PubMed, EMBASE, and Cochrane Library. Keywords used included “traumatic muscle injury”, “immune cells”, “muscle regeneration”, “neutrophils”, “macrophages”, “T lymphocytes”, “regulatory T cell”, “B lymphocytes”, “eosinophils”, “basophils”, “inflammation”, “fibrosis”, “satellite cells”, “immunomodulation”, and “biomaterials”. Priority was given to studies published from 2015 to the present. Emphasis was placed on preclinical models and clinical studies involving humans or rodents. Articles focusing on non-traumatic muscle diseases (e.g., genetic myopathies) were excluded. Also excluded were articles lacking original data on immune cell mechanisms.

Neutrophils constitute the first line of immune defense following TMI, rapidly infiltrating the damaged tissue to clear necrotic debris and potential pathogens. They initiate the inflammatory phase through the release of pro-inflammatory cytokines, including interleukin-6 (IL-6) and tumor necrosis factor-α (TNF-α), and by recruiting circulating monocytes to the injury site. However, excessive or prolonged neutrophil activity intensifies tissue damage, underscoring the importance of tightly regulated inflammatory responses (19). Following neutrophil infiltration, macrophages transition from pro-inflammatory M1 phenotypes to anti-inflammatory M2 phenotypes. M1 macrophages facilitate the clearance of cellular debris and secrete inflammatory mediators, such as TNF-α and IL-1β. M2 macrophages promote tissue regeneration through the release of growth factors including transforming growth factor-β (TGF-β) and IL-10, and support neovascular post-traumatic (20). This macrophage polarization is essential for effective muscle fiber regeneration and vascular remodeling. T lymphocytes, particularly cluster of differentiation 8 positive T cells (CD8^+^ T cells) and regulatory T cells (Tregs), further modulate the immune microenvironment (21). CD8^+^ T cells enhance macrophage recruitment by inducing monocyte chemoattractant protein-1 (MCP-1) expression (22–24), while Tregs attenuate excessive inflammation and facilitate satellite cell activation (25). Other immune cell types, such as eosinophils and basophils, contribute indirectly by secreting cytokines such as IL-4 and IL-13, which regulate fibroblast unction and extracellular matrix remodeling (26–30). The immune response to TMI entails a highly coordinated interaction among immune cells, mediated by cytokines such as high-mobility group box 1 protein (HMGB1), interleukin-33 (IL-33), and other various damage-associated molecular patterns (31). Disruption of this immunological balance may result in persistent inflammation or immunosuppression, hindering tissue regeneration and functional recovery (32–35). Understanding these mechanisms is significant for developing targeted therapies to optimize muscle repair (36).

Despite recent advances, significant challenges remain in translating immune modulation strategies into clinical applications (37). Firstly, the temporal and spatial dynamics of immune cell phenotypes are not fully elucidated, especially in aging populations where macrophage polarization capacity is reduced (38). Secondly, current surgical treatments for Grades III-IV injuries frequently fail to restore native muscle architecture, highlighting the need for combinatorial approaches involving cytokine-targeted biologics, such as anti-TGF-β antibodies (39) and biomaterials engineered to emulate macrophage-derived Wnt7a signaling (40). Novel interventions, including inhibition of neutrophil extracellular traps (NETs) to mitigate chronic inflammation (41) and chimeric antigen receptor - regulatory T cells (CAR-Treg cell) therapies aimed at fibrosis suppression, show promise in preclinical models but require thorough validation in clinical trials (42).

## Etiology and clinical symptoms of TMI

TMI generally result from either indirect non-contact mechanisms, such as strains and fractures, or direct contact injuries, including contusions and lacerations, often caused by mechanical forces and frequently associated with sports-related trauma (1, 43). These injuries generally occur when a muscle is overexerted or subjected to sudden mechanical shock (44), frequently linked to the overstretching of muscle fibers. Moreover, the deformation and ensuing failure of the cell membrane are key components of mechanical injury. Studies suggest that the cell membrane may experience transient rupture following deformation, a process commonly referred to as mechanical porosity (45).

After TMI, just as shown in **Table 2**, varied signs may arise, with pain and discomfort being particularly common, especially following unaccustomed or intense physical activity. This response is attributed to the release of pro-inflammatory mediators by immune cells, which sensitize nociceptors and subsequently induce pain (46). The pain may be immediate or delayed for several days and is often associated with microscopic muscle fiber tears, commonly referred to as delayed onset muscle soreness (46, 50, 51). Muscle stiffness and a temporary reduction in strength are additionally frequent, leading to limited movement after exercise (47). This weakness is primarily caused by structural damage to the muscle and the inflammatory response (52), involving protein degradation, apoptosis, and inflammation-induced ultrastructural damage that impairs muscle function (53). Muscle functionality may be compromised, including a range of motion, coordination, and balance, may be compromised (48). Swelling is another important indicator, visible either locally or systemically, especially after eccentric contraction exercises (54–56). The swelling observed is primarily due to the recruitment of immune cells to the injured site, which increases vascular permeability, facilitating the extravasation of plasma proteins and fluids into the surrounding tissue. These evens collectively reflect the interplay between structural damage and immune-mediated repair mechanisms in response to TMI.

**Table 2:** Clinical symptoms of TMI.

Symptoms	Causes	A link to the immune system	References
Pain and discomfort	Tearing of muscle fibers	Immune cells release pro-inflammatory mediators that cause sensitization of nociceptors, which triggers pain.	(46)
Muscle stiffness	Pain and limited movement after exercise	Inflammation can lead to muscle stiffness, as well as decreased function, etc., because protein degradation, apoptosis, and local inflammatory response during inflammation can lead to ultrastructural damage of muscle cells, resulting in muscle being unable to perform normal function.	(47, 48)
Muscle dysfunction	Damage to muscle structure and inflammatory response		
Swelling of muscle	Inflammation and increased vascular permeability	Immune cells are recruited to the injury site, resulting in increased local vascular permeability, and the infiltration of proteins and other liquids in plasma into the tissue space causing local edema or swelling.	(49)

TMI leads to significant changes in numerous markers, including increased activity of biochemical indicators such as serum creatine kinase (CK), a key marker for assessing the extent of muscle injury (57). Elevated CK levels indicate cell membrane damage and the release of intracellular components (58, 59). Oxidative stress markers, such as malondialdehyde (MDA) and thiobarbituric acid reactive substances, are elevated following injury, reflecting increased levels of oxidative stress (59–61). The immune system releases inflammatory mediators following TMI and modulates immune cell activity. This is reflected in alterations in the number of white blood cells and lymphocytes, which are likely associated with immune activation (62). Research has demonstrated a strong relationship between changes in biochemical markers and the functional status of the immune system (19). For example, serum adenosine deaminase (ADA) activity is elevated following muscle injury, indicating immune system activation. Furthermore, a post-injury immunosuppressed state may arise due to a subsequent decline in immune function (63).

The onset and progression of these responses are influenced by multiple factors, including the type, intensity, and duration of exercise, as well as the individual’s adaptive capacity to physical activity. Early detection of these responses is essential for timely diagnosis and intervention in traumatic muscle injuries, thus facilitating recovery and reducing the risk of further tissue damage.

## Diagnosis and treatment of TMI

Based on these symptoms, a preliminary diagnosis can be made, further supported by diagnostic tools such as ultrasound (64, 65) and MRI (66). These imaging techniques are commonly used to identify the location, extent, and severity of the injury (67, 68). Biomechanical and molecular biological indicators, such as the rate of force development, serve as more sensitive indirect markers for assessing TMI than maximum voluntary isometric contraction peak torque (69).

Treatment for TMI involves several approaches, including initial management, non-surgical and surgical treatments, rehabilitation, and preventive measures. Initial management typically involves protection, rest, optimal use of the injured limb, and cold therapy, following the RICE principle of “rest, ice, compression, and elevation” (70). For more severe injuries, immobilizing the limb for a few days may be necessary to facilitate scarring. Most muscle injuries recover well with conservative, non-surgical treatments, such as non-steroidal anti-inflammatory drugs (NSAIDs) (71), ultrasound therapy (64, 65), strengthening and stretching training (72), and painless joint mobility exercise (1, 9). Surgical treatment is reserved for cases of complete muscle tears or total loss of function (73, 74). Procedures may include hematoma drainage (75) and muscle-tendon reattachment and reinforcement (76). The recovery phase commences after initial treatment, emphasizing rehabilitation to restore function rather than merely alleviating symptoms (77). Primary rehabilitation objectives encompass accurate diagnosis, mitigating the adverse local effects of acute injury, promoting proper healing, preserving motor function, and restoring normal activity levels (78, 79).

Preventive measures are crucial for reducing the risk of muscle injuries, especially in athletes involved in sports such as football. Key strategies include adequate warm-ups, temperature regulation, and stretching, all of which help lower the risk of strains. Increasing muscle strength, endurance, and flexibility is vital for improving athletic performance and preventing injuries. Although traumatic muscle injuries are prevalent among athletes, successful recovery is attainable through proper classification, prompt diagnosis, and effective treatment.

## Immune cells response to TMI repair

After TMI, the immune system initiates an inflammatory response to remove damaged cells and tissue debris, setting the stage for muscle tissue repair. This process involves vasodilation, cell migration, and the activation of inflammatory cells (80). Simultaneously, the immune system orchestrates and regulates the functions of diverse cellular components, with macrophages playing a central role in driving muscle tissue repair and regeneration following TMI (81). After an injury, the immune system activates muscle stem cells, transitioning them from a quiescent state to active proliferation, which forms the basis for effective muscle tissue repair and regeneration (82). The immune system induces fibroblasts to differentiate into myofibroblasts, which produce collagen and other extracellular matrix components vital for providing structural support to the newly formed muscle tissue (83). It also promotes angiogenesis, ensuring a sufficient nutrient supply and oxygenation for the regenerating muscle tissue.

Moreover, the immune system orchestrates the activities of immunocompetent cells such as macrophages, lymphocytes, and dendritic cells, which secrete multiple cytokines to enhance muscle repair and regeneration (80). The interaction between the immune system and muscle repair is highly coordinated, involving the recruitment and functional transformation of immune cells and other key processes. Continued research in this area improves our understanding of muscle repair mechanisms and may provide novel strategies for treating related disorders.

TMI triggers an intricate host response that disrupts immune homeostasis, increasing susceptibility to opportunistic infections and inflammatory complications such as infections, multiple organ dysfunction syndrome (MODS) (84), immunosuppression (85), and associated inflammatory responses. Following trauma, the body initiates a cascade of immune reactions to control inflammation at the injury site and support tissue repair (86). However, excessive production of pro-inflammatory cytokines can drive the immune response into an immunosuppressive state (86), significantly contributing to complications like nosocomial pneumonia, systemic inflammatory response syndrome, and MODS (86). Severe trauma often leads to increased activation of innate immune cells and overproduction of inflammatory mediators (87). This over-activation can result in systemic pro-inflammatory circulation and cytokine storms (88) that provoke strong non-infectious systemic inflammatory responses (89). MODS can arise from an orchestrated inflammatory response (90). These observations underscore a crucial point: the complications are closely linked to the immune system, and the regeneration of TMI relies heavily on the inflammatory processes during this phase (91). Furthermore, immune cells are essential in coordinating the inflammatory response, initiating transient inflammation following various types of injury (92). The development and function of these cells are modulated by proteins whose expression is influenced by the surrounding inflammatory environment (93–95). In summary, most complications from traumatic muscle injuries are closely associated with inflammation, primarily driven by immune responses, including activating pro-inflammatory factors and immune cell participation.

**Figure 1:**
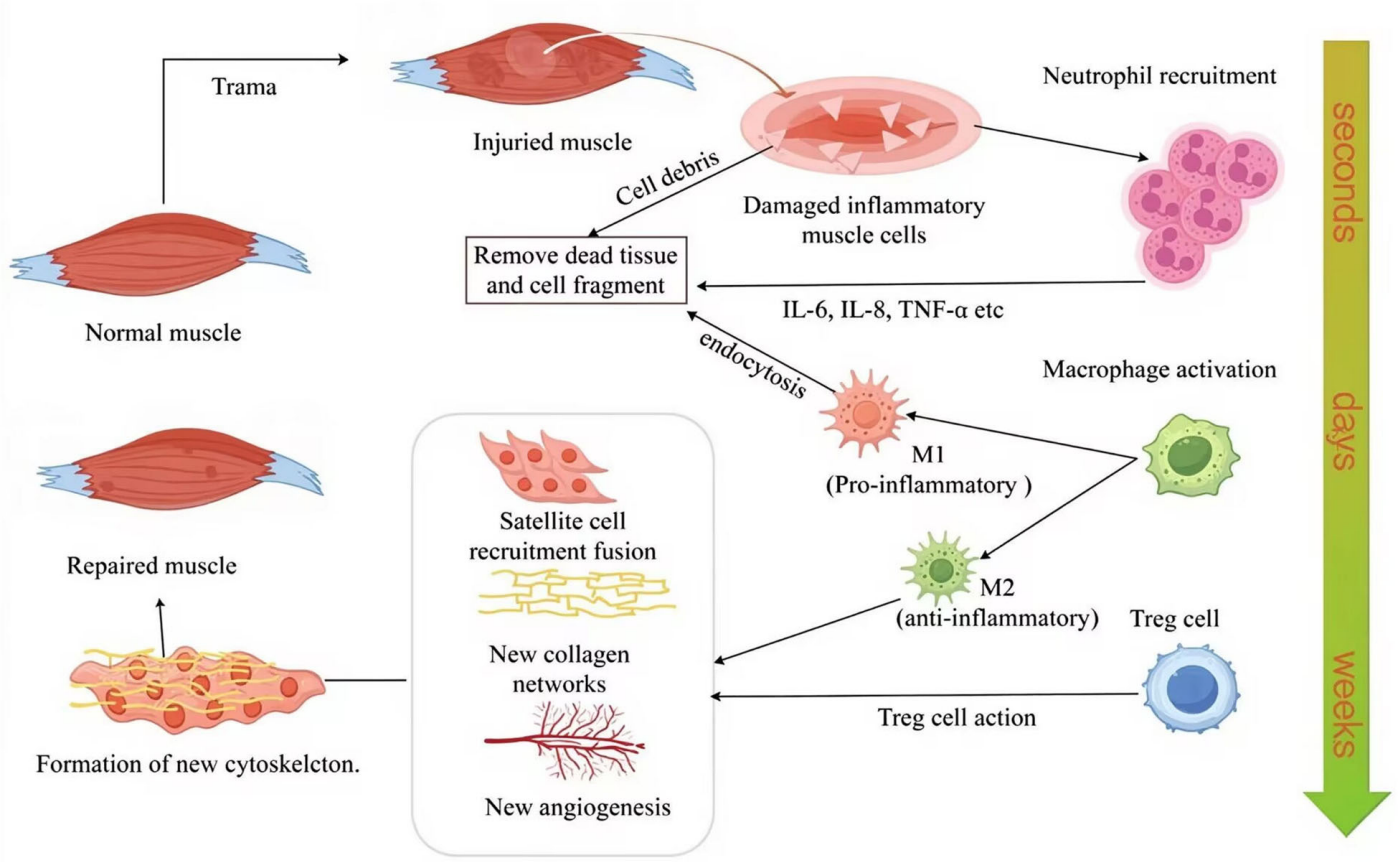
Immune cell dynamics during TMI repair, including, neutrophils immediately infiltrate within a few seconds, macrophage polarization in a few days, and Treg-mediated inflammation regulation in weeks. M1, pro-inflammatory macrophage; M2, anti-inflammatory macrophage; IL-6, interleukin-6; IL-8, interleukin-8; TNF-α, tumor necrosis factor-α; Treg cell, regulatory T cell. The figure was drawn by Figdraw.

As shown in **Figure 1**, the healing process following TMI progresses through distinct phases, beginning with an inflammatory response. Upon muscle damage, resident cells within the affected tissue release a spectrum of cytokines, including pro-inflammatory mediators such as TNF-α and IL-1β, as well as anti-inflammatory cytokines such as IL-6, interleukin-8 (IL-8), and interferon-γ (IFN-γ). These cytokines upregulate adhesion molecule expression, facilitating the recruitment of leukocytes from the circulation to the injury site. Neutrophils are the first immune cells to infiltrate the damaged tissue, where they play a pivotal role in removing necrotic material and cellular debris, initiating the repair process (96, 97). As the process advances, macrophage activation shifts significantly, with pro-inflammatory macrophages dominating the local microenvironment. These macrophages secrete pro-inflammatory cytokines, further promoting myogenic cell proliferation and expanding the satellite cell population. Depending on their activation phenotype, macrophages can adopt various roles throughout TMI repair (98). Tregs also play a significant role. Studies have shown that these cells can accelerate healing processes when directly injected into damaged bone, muscle, or skin (99). Tregs regulate immune responses while creating a microenvironment conducive to effective tissue repair (100, 101). Following debris clearance, satellite cell recruitment and fusion occur: quiescent satellite cells become activated and proliferate, then migrate to the site of injury, where they fuse with adjacent muscle fibers to form new myofibers, ultimately restoring muscle function (102). Finally, new vascular networks form during the fibrosis and maturation phases, and collagen deposition occurs, driven by factors released from macrophages as healing advances. The process culminates in the maturation stage, where damaged tissues are either fully replaced or remodeled to restore functional normalcy (103).

The immune system’s response to TMI involves a multifaceted interplay of mechanisms mediated by immune cells, which are crucial for post-injury muscle repair. Following trauma, these cells play pivotal roles, including the activation and differentiation of muscle stem cells, the regulation of inflammation, the clearance of damaged tissues, and active participation in tissue regeneration.

## Neutrophils

Neutrophils play an important role in both the initial injury and repair of TMI, serving as an necessary component of the innate immune system that combats infections through pathogen engulfment and the release of antibacterial enzymes (104). In both murine and human studies, an inflammatory response is triggered upon TMI, producing diverse inflammatory cytokines and chemokines (105). These substances, such as IL-6, IL-8, C-X-C motif chemokine ligand (CXCL) neutrophil chemokines, and TNF-α, act on endothelial cells adjacent to blood vessels, attracting and recruiting neutrophils to the site of injury (106, 107). Many members of the CXCL family, as potent neutrophil chemokines, are secreted by various cell types such as endothelial cells, fibroblasts, and macrophages (108). They induce neutrophil polarization and migration by activating CXCR1 and CXCR2 receptors on neutrophils (109). Moreover, they form a concentration gradient near the injury site, guiding neutrophils to migrate from the intravascular space to the damaged area. Endothelial cells play a crucial role in this process. They not only secrete chemokines but also upregulate the expression of adhesion molecules (e.g., selectins) to promote neutrophil rolling and attachment (110). Additionally, upon treatment with IL-1 or TNF-α, endothelial cells can synthesize and secrete chemokines structurally similar to the neutrophil-activating factor (NAF) derived from human monocytes, further enhancing neutrophil activation and migration (111). Cytokines such as IL-6 and TNF-α also play important roles in the inflammatory response; they can stimulate endothelial cells to produce more chemokines, thereby strengthening neutrophil recruitment (112). At the very beginning of TMI, neutrophils rapidly migrate to the injured area to eliminate necrotic tissue and cellular debris, creating favorable conditions for reparative processes. Although the core mechanisms of neutrophil recruitment and function are conserved between mice and humans, species-specific differences in cytokine kinetics or receptor expression may affect the magnitude and duration of their response (113).

During the early stages of TMI, neutrophils contribute to the inflammatory response by releasing reactive oxygen species (ROS) and proteases, which facilitate the initial repair of the injured site (114, 115). NETosis (Neutrophil Extracellular Trap formation), a mechanism by which neutrophils release NETs during infection or inflammation to capture and kill pathogens through reticular structures, also contributes here (116, 117). Removing cell and fiber debris facilitates muscle regeneration and connective tissue deposition and triggers a strong inflammatory response (105). As a result, the over-activation of neutrophils may lead to further tissue damage, highlighting the need to precisely regulate their function (118). However, recent studies highlight the functional plasticity of neutrophils, with pro-inflammatory N1 and anti-inflammatory N2 phenotypes mirroring the M1/M2 macrophage dichotomy (119). In the acute inflammatory stage, N1 neutrophils are dominant and release reactive oxygen species and proteases, such as matrix metalloproteinase-8 (MMP-8) and MMP-9, which are involved in the killing of pathogens (120). During the inflammation resolution phase, N2 neutrophils emerge and express anti-inflammatory molecules including CD206, Arg1, and IL-10, thereby participating in tissue repair and scar formation (121). Furthermore, N2 neutrophils, which appear during the resolution phase of inflammation, secrete anti-inflammatory mediators such as IL-10 (122, 123). Notably, under the influence of cytokines such as CXCL1, neutrophils undergo reverse migration from damaged muscle tissue (116). This process rapidly depletes local neutrophil populations, promoting inflammation resolution and creating a favorable microenvironment for tissue regeneration and repair (116). Reverse-migrated neutrophils re-enter the vasculature, transit through the lungs, and return to the bone marrow for clearance (116). This clearance pathway represents the most favorable outcome for effective tissue repair. However, aging significantly impacts this process. The N3 ageing signatures impacts muscle function, including changes in muscle mass, fiber type, and reduced regenerative capacity of muscle stem cells (124). Additionally, age alters immune dynamics post-traumatic muscle injury (TMI). Compared to young mice, older mice exhibit delayed neutrophil clearance after peak infiltration and show age-specific changes in monocyte/macrophage subpopulation abundance (125).

As the inflammatory response at the injury site gradually subsides, IL-10 from N2 neutrophils promotes macrophage polarization toward a regenerative phenotype, enhancing myoblast proliferation and satellite cell activation in murine models (126–128). However, human studies suggest additional complexity, with neutrophil-derived extracellular vesicles (EVs) playing a role in satellite cell regulation (129). Moreover, anti-inflammatory macrophages release pro-inflammatory cytokines such as IL-6 and TNF-α, which stimulate myoblast proliferation and satellite cell recruitment. These cells then fuse with surrounding muscle fibers, forming new myonuclei, enhancing protein synthesis, and promoting muscle regeneration (73, 130, 131). It is important to note that cytokines and chemical signals finely regulate neutrophil function following muscle injury. IL-8 facilitates neutrophil recruitment and function, while TNF-α is crucial in initiating early inflammatory responses (119, 132). During muscle repair, these cytokines not only influence neutrophil migration and function but exert downstream effects on other immune cells, including macrophages and lymphocytes, during muscle repair.

In summary, neutrophils show dual functions in TMI repair. They facilitate initial repair by eliminating necrotic tissue and pathogens, while promoting muscle regeneration through the release of pro-inflammatory factors and participation in the inflammatory response (104, 115, 133). However, their over-activation can exacerbate tissue damage, necessitating precise regulation of their function for optimal repair (115).

Studies in murine and human models underscore neutrophils’ pivotal yet complex role of neutrophils in muscle regeneration (41, 134). In studies on physical therapy for damaged muscles using mouse models, David J. Mooney’s team conducted a series of experiments demonstrating that massage therapy can facilitate the removal of neutrophils from injured muscles. This reduction minimizes the negative impact of neutrophil-associated secretions on muscle progenitor cell differentiation, promoting myogenesis by influencing muscle fiber maturation and supporting muscle regeneration (135). Furthermore, studies in CCR2-deficient mice have shown that the peak number of neutrophils coincides with the activation of muscle satellite cells, suggesting that neutrophils may play a role in satellite cell activation (136). This is critical because satellite cells are necessary for maintaining uninjured muscle and rapidly responding to growth or regeneration signals to re-enter the cell cycle (137, 138). Neutrophils influence the dynamics of satellite cells by releasing paracrine factors, further supporting their involvement in muscle regeneration (139). The heterogeneity and plasticity of neutrophils contribute to tissue repair. They clear damaged tissue, and form NETs, which aid in regulating cell proliferation (140, 141). Finally, neutrophil depletion studies, such as those involving mice injected with snake venom toxin, have demonstrated that the absence of neutrophils leads to significant tissue necrosis and impaired regenerative responses (1, 142).

Other studies have suggested that neutrophils may not significantly impact muscle recovery. In one study, mice underwent a 10-day offloading of their hind limbs, followed by reloading after neutrophil depletion. The results indicated that neutrophil depletion did not affect strength loss or the recovery of atrophied muscle fibers (143). The potential for neutrophils to exacerbate muscle injury has been extensively explored, as summarized in **Table 3**. Besides the previously reported reduction of neutrophils through massage therapy to promote muscle repair (135), and hyperactive neutrophils contribute to severe inflammation. Neutrophil-derived mediators such as superoxide dismutase 2 (SOD-2), glutathione peroxidase (GPX), catalase (CAT), and thioredoxin (TRX) induce muscle fiber damage, membrane disruption, and oxidative lipid degradation (146–148). For instance, myeloperoxidase (MPO), a key enzyme in neutrophils, generates ROS such as hypochlorous acid, which plays a crucial role in microbial killing but can also contribute to tissue damage (145).

**Table 3:** Overview of the role of neutrophils in TMI repair.

Reference	Research model/platform	Research mechanism	Conclusions related to immune cells
(136)	Dual laser Multimode nonlinear optical microscope platform	The peak number of neutrophils coincided with the activation of muscle satellite cells.	Neutrophils may be involved in the activation of satellite cells in favor of muscle repair.
(142)	Male Swiss mice were pretreated with anti-mouse granulocyte immunoglobulin G (IgG) or control antibody.	Likely through phagocytosis of necrotic debris and the recruitment of other inflammatory cells, both of which are critical for effective muscle repair.	Neutrophils play a key role in skeletal muscle regeneration following Bothrops asper.
(144)	A peptidyl arginine deiminase 4 (PAD4) deficient mouse model	Neutrophils induce secondary immune thrombosis through PAD-dependent mechanisms, thereby promoting the healing of injured tissues.	Insufficient immune thrombosis caused by neutrophils may cause damaged tissue to bleed and be difficult to heal.
(143)	Mice underwent 10 days of hindlimb unloading and neutrophil depletion before reloading.	LPS alters the activation state of neutrophils, otherwise, neutrophilic infiltration is highly regulated and effectively eliminated during regulated mechanical loading without significant muscle fiber damage	Neutrophil consumption does not affect loss of strength or restoration of atrophied fibers.
(135)	Real-time force control compatible with ultrasound for tissue strain analysis.	In addition to neutrophils in the injured muscle, the inhibitory effect of neutrophil-related secretion factors on the differentiation of muscle progenitor cells was reduced.	Massage to clarify that neutrophils promote myogenesis by changing the type of muscle fiber maturation, thus promoting muscle regeneration.
(145)	Humans normally heal open damage and pressure ulcers.	MPO deficiency leads to an intensification of the inflammatory response and affects neutrophil function, including cytokine production.	MPO, as a key enzyme in neutrophils, produces reactive oxygen intermediates such as hypochlorous acid that help kill microorganisms, but may also cause tissue damage.
(146)	Nanoparticles were used to label bone marrow-derived mesenchymal stem cells (BM-MSCs)	Neutrophil-derived mediators are made by SOD. When hydrogen peroxide accumulates, it is toxic to cells, causing membrane lipid peroxidation and membrane fission, leading to cell damage and death.	When the medium produced by neutrophils is less superoxide, the cytotoxicity and the influence on cell proliferation activity are less, which is conducive to tissue repair.

The presence and activity of neutrophils may be associated with the pathogenesis of TMI. Animal studies have shown that neutrophil depletion can enhance muscle regeneration, supporting the hypothesis that neutrophils contribute to muscle damage. Researchers found the depletion of neutrophils in young mdx mice reduced muscle degradation (149). Furthermore, in the mdx animal models of muscular dystrophy, elevated levels of neutrophil elastase and ROS have been shown to impair myoblast survival and differentiation (150). Overstimulation of neutrophils in these contexts leads to excessive NETs formation, which is cytotoxic and can hinder muscle tissue regeneration; studies have shown that the components of NETs, namely purified histone type-IIA, can inhibit cell growth in a concentration-dependent manner and induce cytotoxicity. This thereby hinders the repair of various tissues (151).

Neutrophils play diverse roles in TMI repair, including initiating the inflammatory response, facilitating muscle regeneration, modulation of the inflammatory environment, and contributing to dual functions. In the pathophysiological process of TMI, neutrophils play a complex and bidirectional regulatory role (115). Their functions exhibit significant duality - they not only provide necessary support for early inflammation initiation and tissue regeneration, but also may exacerbate secondary damage due to excessive activation (104). From a positive perspective, after injury occurs, neutrophils rapidly migrate through the vascular endothelium to the damaged muscle tissue, using phagocytosis to clear local necrotic muscle fiber fragments and invading pathogens, creating a microenvironment for subsequent repair (109, 116). At the same time, the proteins and ROS secreted by them can activate satellite cells through signal transduction pathways, promoting the proliferation and differentiation of myogenic precursor cells; the released chemokines (such as CXCL8) can recruit monocytes and macrophages to infiltrate the injury site, initiating the regeneration cascade reaction. Moreover, NETs play a key role in limiting the expansion of the injury range by capturing pathogens and degrading cell debris, thereby restricting oxidative stress damage to healthy muscle fibers and degradation of extracellular matrix. However, excessive activation and continuous infiltration of neutrophils may lead to amplification of the injury effect (117). Excessive ROS and proteins can exceed the physiological regulatory threshold, directly causing oxidative damage to healthy muscle fibers and degradation of the extracellular matrix. This inhibits the regenerative potential of muscle satellite cells; moreover, the pro-inflammatory mediators these cells secrete delay the phenotypic transformation of macrophages from M1 to M2, hinder the establishment of an anti-inflammatory microenvironment, and thereby promote fibrosis and ectopic ossification (116). In the future, through precise regulation of their temporal and spatial dynamics, while retaining their early clearance function, and avoiding excessive damage, it will be possible to maximize their promoting effect on muscle repair (152).

## Monocytes/macrophages

Macrophages are critical to TMI, which is pivotal in initiating and resolving inflammatory responses (153, 154). Their functions are regulated through distinct phenotypic polarization—M1 and M2—which either promote or inhibit muscle regeneration (155, 156). Herein, there are three main approaches to macrophage differentiation: 1) each monocyte subpopulation differentiates into a specific macrophage phenotype; 2) the macrophage phenotype is determined by microenvironmental signals and cytokines, primarily in the context of inflammation; 3) mature macrophages can shift between pro-inflammatory (M1) and anti-inflammatory (M2) phenotypes in response to changes in their tissue environment (157, 158).

**Figure 2:**
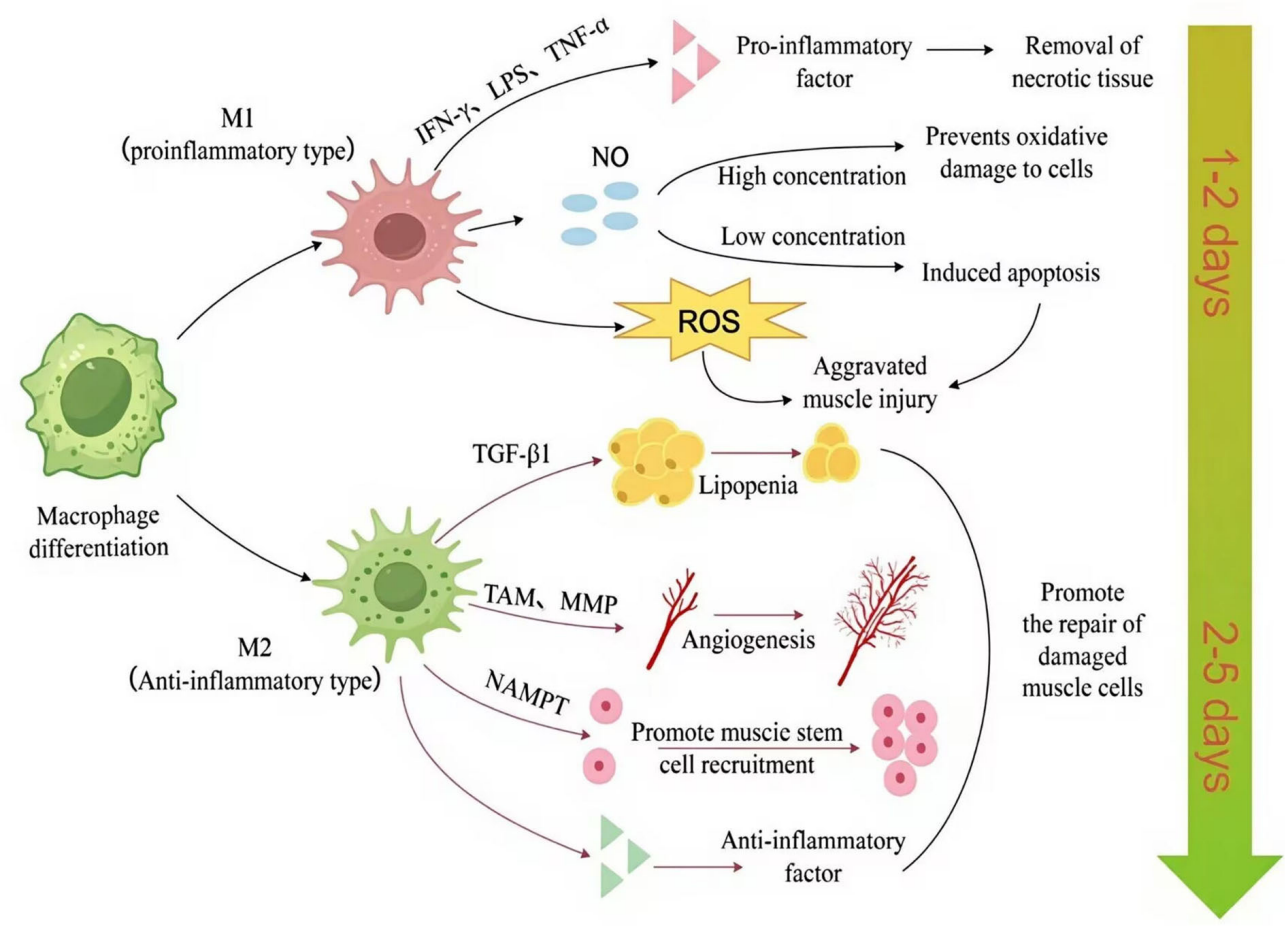
Macrophages shift from a pro-inflammatory (M1) phenotype (peaking at 0.5–2 days post-injury) to an anti-inflammatory (M2) phenotype (maximal at 3.5–10 days), dynamically coordinating tissue debridement, stem cell activation, and regenerative remodeling (2). The figure was drawn by Figdraw. ROS, reactive oxygen species; TAM, tamoxifen; MMP, matrix metalloproteinase; NO, nitric oxide; NAMPT, nicotinamide phosphoribosyl transferase. TGF-β1, transforming growth factor-β1; LPS, lipopolysaccharide; TNF-α, tumor necrosis factor-α; IFN-γ, interferon-γ; M1, pro-inflammatory macrophage; M2, anti-inflammatory macrophage.

M1 macrophages, primarily pro-inflammatory, contribute to clearing damaged tissue by engulfing apoptotic or necrotic muscle fibers, releasing inflammatory mediators such as TNF-α and IL-1β (159, 160), and activating other immune system components. These pro-inflammatory actions facilitate tissue cleaning and fibrous debris (139). However, as clearly illustrated in **Figure 2**, M1 macrophages may also intensify muscle damage by generating ROS and nitric oxide (NO) (161). NO concentration critically determines its effect: high levels induce apoptosis, while low levels protect against oxidative stress and even promote muscle cell proliferation/growth in early repair (162, 163), suggesting NO modulates the regeneration-fibrosis balance. Furthermore, prolonged M1 activation promotes excessive fibrosis (164, 165) and can inhibit axon (166, 167) and potentially muscle fiber regeneration (168) impairing long-term recovery. In summary, while M1 macrophages are essential for initial inflammation and clearance, their overactivation or persistence negatively impacts repair through fibrosis and inhibition of regeneration (169, 170). Meanwhile, studies have shown that by synthesizing PLGA (poly(lactic-co-glycolic acid)) nanoparticles that encapsulate magnesium and delivering them to macrophages to reduce the M1 phenotype, the macrophage phenotype can be resolved in the context of muscle repair to alleviate inflammation and improve tissue regeneration (171). In summary, M1 macrophages release pro-inflammatory factors that serve a dual function in muscle repair.

In contrast, M2 macrophages transition from an initial anti-inflammatory role to become central mediators of tissue regeneration. This functional adaptation is facilitated by distinct M2 subtypes (M2a, M2b, M2c, M2d), classified based on activating stimuli and transcriptional profiles, each with specific functions (172). M2a is activated by IL-4 or IL-13 and is primarily involved in immune regulation and tissue repair. M2b is activated by LPS or IL-1β and has immune regulatory and inhibitory functions. M2c is activated by IL-10, TGF-β, or glucocorticoids and exhibits anti-inflammatory and immunosuppressive properties. M2d is activated by stimuli such as IL-10, IL-12, TNF-α, TGF-β, Vascular endothelial growth factor (VEGF), and MMPs, and its main functions are immunosuppression and angiogenesis.

As regeneration progresses into later stages, M2a macrophages, also referred to as alternatively activated macrophages, predominantly function during the early stages of immune responses. They suppress inflammatory reactions and stimulate the proliferation of non-myeloid cells through the secretion of regulatory cytokines such as IL-4 and IL-13. Additionally, M2a macrophages play a role in fibrosis by regulating fibroblast activities via the secretion of factors like TGF-β (173). M2b macrophages show increased activity during the later stages of muscle regeneration. They facilitate the proliferation and differentiation of muscle cells while simultaneously reducing apoptosis, and improving enhancing muscle repair capacity. Evidence suggests that M2b macrophages promote muscle cell differentiation and muscle fiber formation through the secretion of anti-inflammatory factors such as TGF-β1 (91). Furthermore, M2b macrophages suppress the activity of M1 macrophages and mitigate inflammatory responses, thus fostering an environment conducive to muscle regeneration. M2c macrophages inhibit the pro-inflammatory function of M1 macrophages by secreting anti-inflammatory factors such as IL-10 and TGF-β, facilitating tissue repair and fibrosis processes. They can inhibit the phenotype of M1 macrophages and promote the proliferation of non-myeloid cells. M2c macrophages are likewise involved in the fibrosis process and promote tissue remodeling by regulating the differentiation of fibroblast precursors (11).

Following skeletal muscle injury, macrophage subsets. Spatially and Temporally form multi-layered regenerative inflammatory zones (RIZs). Spatially resolved transcriptomic analyses reveal that the dynamic changes in macrophage subtypes and the ordered structure of RIZs are fundamental to efficient skeletal muscle regeneration (174). Within these zones, subsets such as Growth Factor expressing macrophages (GFEMs) promote regeneration by secreting factors like growth differentiation factor (GDF-15), a process transcriptionally controlled by the PPARy/RXR.axis GDF-15 deficiency. results in delayed muscle regeneration,. highlighting. GFEMs and GDF-15 as key regulatory factors in regenerative repair (175).

Exploring the specific mechanism of macrophages in TMI, we know macrophages are modulated by multiple signaling pathways during their polarization and functional transformation. Following tissue injury, the nuclear factor-κB (NF-κB) pathway is activated by Toll-like receptors (TLRs) (176), ROS, or damage-associated molecular patterns (DAMPs) (177). This activation dominates the pro-inflammatory response and regulates the release of inflammatory cytokines such as TNF-α. While this process facilitates the clearance of necrotic tissues during the acute phase, persistent activation may lead to chronic inflammation. The p38 branch of the mitogen-activated protein kinase (MAPK) pathway enhances the secretion of pro-inflammatory factors, including TNF-α, and augments phagocytic activity (178). In comparison, the extracellular signal-regulated kinase (ERK) branch is associated with the production of the anti-inflammatory cytokine IL-10 and may play a role in the repair phase. The c-Jun N-terminal kinase (JNK) branch mediates apoptotic signals and amplifies inflammatory responses (179). Within the signal transducer and activator of the transcription (STAT) family, STAT1 promotes the expression of pro-inflammatory genes upon activation by IFN-γ, whereas STAT3 attenuates inflammation by inhibiting NF-κB under the influence of IL-10 signaling. Additionally, STAT6 induces the expression of anti-inflammatory and repair-related genes in response to IL-4/IL-13 stimulation (180). The TGF-β/Smad pathway regulates collagen synthesis and myofibroblast differentiation by suppressing inflammation and promoting fibrosis (181). In the phosphoinositide 3-kinase/protein kinase B/mammalian target of rapamycin (PI3K/Akt/mTOR) pathway, protein kinase B activation inhibits NF-κB activity and stimulates the secretion of anti-inflammatory factors, while target of rapamycin complex 1 (mTORC1) influences macrophage function through metabolic reprogramming, thereby maintaining a balance between inflammation and repair (179).

Recent studies have shown the effect of macrophages on the behavior of myoblasts and muscle stem cells by releasing paracrine factors. For example, real-time imaging with genetically modified zebrafish has allowed scientists to observe interactions between muscle stem cells and the innate immune system, underscoring the critical role of macrophages in muscle regeneration (136). Studies have further demonstrated that macrophages are rapidly activated by the complement system during muscle injury, particularly through the complement activation molecule complement component 3a (C3a). This molecule recruits macrophages via its receptor, C3a receptor, and plays a pivotal role in regeneration following skeletal muscle damage (182). The metabolic state of macrophages is another key factor influencing muscle regeneration. Sterol regulatory element binding protein 1 has been identified as a regulator of macrophage lipid metabolism, which affects their ability to facilitate tissue repair. Macrophages aid in the activation and proliferation of muscle stem cells by secreting specific factors that promote differentiation and fusion of these cells, improving muscle regeneration (183). Research has also identified that muscle stem cell subpopulations labeled with glioma-associated oncogene 1 positive (Gli1^+^) remain in an “alert” state in uninjured muscle, allowing for a rapid and efficient response to injury, thus accelerating muscle repair.

Macrophage activation and function are highly versatile, with distinct roles at different muscle injury and repair stages. In both stable and injured skeletal muscle, their functional diversity encompasses phagocytosis, regulation of inflammation, and tissue remodeling (184).

## Eosinophils and basophils

Both eosinophils and basophils play vital roles in TMI repair. Eosinophils contribute significantly to muscle tissue repair and epithelial remodeling by interacting with the clotting system to promote hemostasis and tissue repair. Moreover, eosinophils are recruited to damaged organs such as the liver and muscles, where they secrete IL-4 and IL-13. These cytokines activate IL-4 receptor α Chain-expressing hepatocytes and progenitors in both liver and muscle tissues, supporting tissue regeneration. IL-4 activates the IL-4Rα/STAT6 signaling pathway on (Fibro adipogenic progenitors) FAPs, thereby promoting the proliferation of FAPs (185–189). Although initially implicated in muscle fat degeneration, heterotopic ossification, and fibrosis, FAPs are now recognized as essential for skeletal muscle homeostasisc (190). Takahashi et al. demonstrated that autocrine IL-33-suppression of tumorigenicity 2 (ST2) signaling in FAPs protects against immobilization-induced atrophy (190). Furthermore, recombinant IL-33 administration counteracted this atrophy in aging murine models (190). Notably, creatine supplementation elevates macrophage ATP levels, which promotes eosinophil recruitment and consequently enhances their antigen presentation, inflammatory responses, and critically, their muscle repair capacity (191).

On the other hand, basophils also play a significant role in immune surveillance and damages repair. Although they constitute only a small fraction of circulating white blood cells, basophils release histamine and other mediators necessary for initiating allergic reactions (192). While the exact mechanisms of basophil action remain incompletely understood, they are believed to be important for maintaining normal physiological functions and responding to trauma. Recent studies suggest that basophils contribute to traumatic muscle repair by releasing multiple mediators. These mediators include some anti-inflammatory substances, such as IL-4 and IL-13; as well as pro-inflammatory substances, such as IL-6, IL-9, CCL8 and granulocyte-macrophage colony-stimulating factor (GM-CSF), all of which play roles in inflammatory responses and tissue repair (193). IL-4, a key mediator released by basophils, can promote the polarization of macrophages toward the M2 phenotype (194). Additionally, IL-4 can indirectly influence the muscle repair process by activating mast cells and eosinophils (194). IL-6 plays a significant role in the inflammatory response; it not only participates in the early inflammatory reaction but also promotes muscle repair by regulating the proliferation and differentiation of satellite cells. Meanwhile, IL-6 may bind to receptors on the surface of muscle cells to enhance their repair and regeneration (165). Similar to IL-4, IL-13 has anti-inflammatory and reparative effects, which can promote the polarization of macrophages toward the M2 phenotype and enhance their phagocytic capacity, thereby clearing damaged tissue debris and creating conditions for muscle repair (195). CCL8, as a chemokine, can attract neutrophils and macrophages to migrate to the injury site, thus accelerating the inflammatory response and tissue repair (196). Granulocyte-macrophage colony-stimulating factor (GM-CSF) can promote the activation and proliferation of macrophages, enhance their phagocytic function, and at the same time stimulate the activity of fibroblasts and promote the synthesis of collagen, thereby supporting the repair of muscle tissue (165). Overall, these mediators promote inflammation by recruiting more immune cells to the injury site, thereby accelerating muscle regeneration. Basophil activation is triggered through type I hypersensitivity reactions and immunoglobulin E (IgE)-mediated type 2 inflammation. When allergens bind to IgE-sensitized mast cells and basophils, these cells degranulate, releasing histamine and further amplifying the inflammatory response. Furthermore, basophils facilitate macrophage activation and migration, and recruit other immune cells by secreting additional pro-inflammatory mediators such as IL-6 and IL-9. This activity aids in clearing damaged tissue and promoting new tissue formation (193). Eosinophilic and basophilic granulocytes repair traumatic muscle through diverse mechanisms, including promoting hemostasis, tissue regeneration, and inflammation regulation.

## T lymphocytes

T lymphocytes, or T cells, are crucial components of the immune system, maturing primarily in the thymus before migrating to various immune organs and tissues to carry out their immune functions. They are derived from pluripotent stem cells in the bone marrow and, during embryonic development, from the yolk sac and liver (197, 198). Studies have shown that T lymphocytes are essential in repairing and regenerating severely injured muscle tissue in mice (100). T lymphocytes accumulate in human muscle tissue following injury and contribute to the “repetitive practice effect”, enhancing muscle resilience against recurrent damage (199). They play a vital regulatory role post-injury. For instance, increased αβ T cells may help suppress inflammation, and activated γδ T cells (expressing CD4/CD69) modulate the inflammatory response (200).

**Figure 3:**
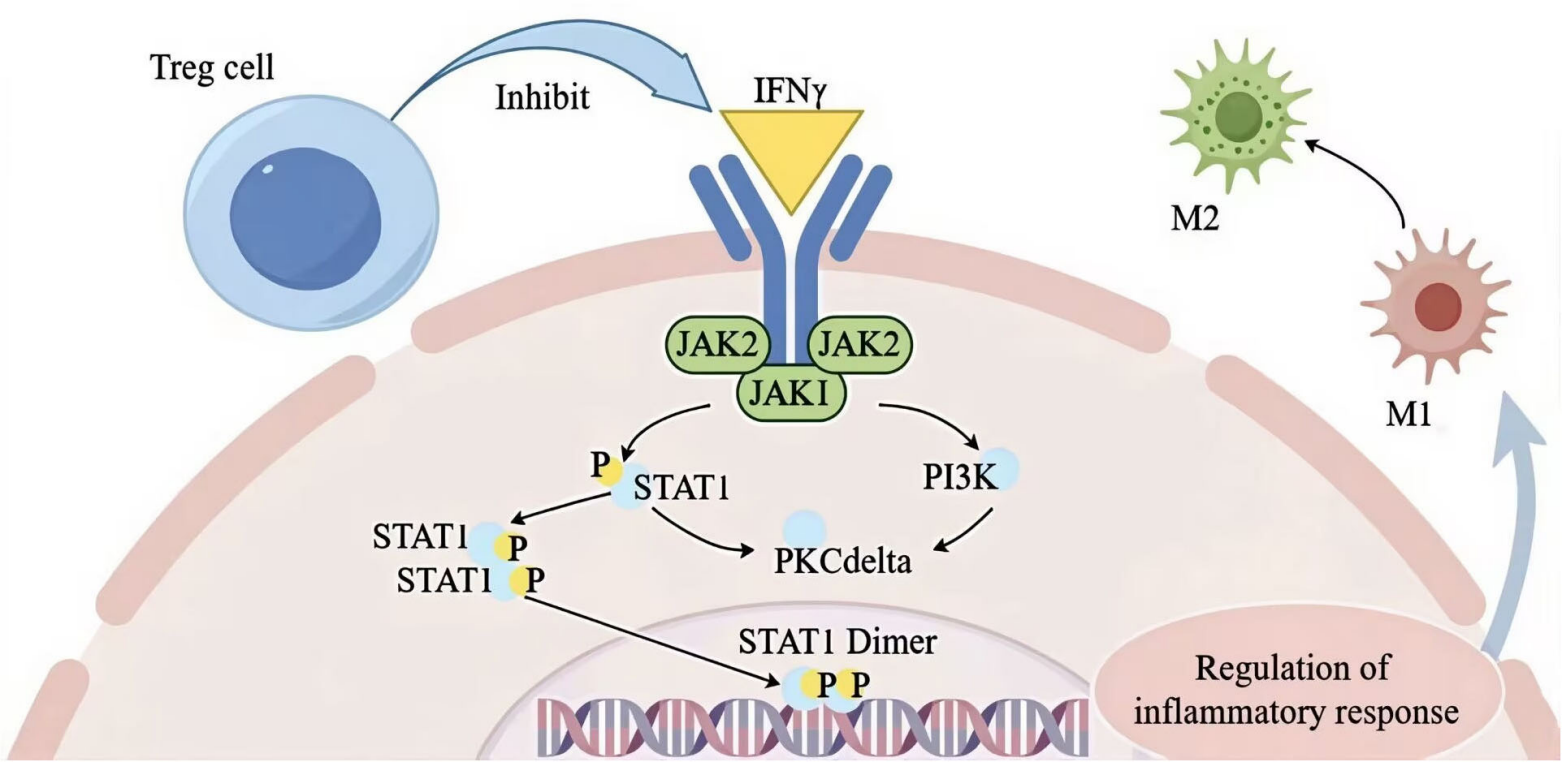
Treg cell modulation of IFN-γ signaling to promote M1-to-M2 macrophage transition and resolve inflammation during muscle repair. The figure was drawn by Figdraw. STAT1, signal transducer and activator of transcription 1; JAK1, janus kinase 1; PKC delta, protein kinase C delta, PI3K phosphatidylinositol 3-kinase; M1, pro-inflammatory macrophage; M2, anti-inflammatory macrophage; IFN-γ, interferon-γ; P, phospholipid.

Investigations into T lymphocytes have highlighted the significant role of Tregs in muscle recovery. Numerous studies have demonstrated that Tregs are essential for maintaining muscle tissue’s homeostasis, integrity, and functionality. They regulate skeletal muscle function and regeneration through IL6 receptor α signaling (201). One study revealed that exercise enhances Treg stability, improving muscle retention and increasing the expression of molecules such as calmodulin, epidermal growth factor receptor (EGFR), and ST2. The functional capabilities of Tregs were shown to restore muscle repair in IL6 receptor α triple knockout (TKO) mice (201). Further research suggests that Tregs produced in the gut can contribute to repairing injured muscles and damaged livers, underscoring the significant influence of gut microbiota on Treg function and their role in muscle healing. The beneficial effects of Tregs on muscle repair can be categorized in several ways. In terms of inflammation regulation, Tregs inhibit the IFN-γ signaling pathway (202), as illustrated in **Figure 3**. This inhibition reduces the expression of associated genes on macrophage cell membranes and promotes the transition from pro-inflammatory M1 to anti-inflammatory M2 macrophages, thereby facilitating muscle repair (203). The regulation of Tregs is crucial for the effective transformation and repair of skeletal muscle inflammation, directly influencing the reparative outcomes of damaged skeletal muscle (204). Therefore, given that Tregs already have a repairing effect in traumatic muscles, and the CAR-Tregs technology enhances the specificity and function of Tregs, enabling them to target specific antigens more precisely and further improving the immune regulatory effect, it thus demonstrates significant clinical application prospects in the treatment of traumatic muscle injuries (42).

Other T cell subsets, such as CD8^+^ T cells, also influence muscle repair processes. It is suggested that the releasing of pro-inflammatory molecules from damaged muscle fibers may exacerbate muscle damage induced by CD8^+^ T lymphocytes (205). Studies have shown that in patients with active juvenile dermatomyositis, a reduction in peripheral circulating CD8^+^ T cells may be linked to specific muscle injury phenotypes (206). Although CD8^+^ T cells do not directly participate in muscle repair, they can regulate the balance between T cell subsets, including Th1, Th2, Th17, and Treg cells, through immune modulation. This regulation affects the differentiation and function of CD4^+^ T cells, ultimately influencing the repair process following TMI (207).

## B lymphocytes

B lymphocytes are key components of the immune system, playing essential roles in antibody production and the establishment of immune memory (208, 209). Studies have highlighted their direct involvement in diseases related to muscle injury-related diseases, particularly inflammatory myositis, whereas their role in traumatic muscle injuries appears more indirect (210). Although B lymphocytes are primarily associated with the humoral immune response, they may indirectly influence cellular immune responses through cytokine secretion and immune regulation. Following trauma, the immune system regulates a multifaceted interplay of inflammatory and regulatory responses. Evidence suggests that trauma can impair B lymphocyte function. For instance, a study found that B cells from trauma patients exhibited significantly reduced immunoglobulin synthesis and secretion *in vitro*, a defect attributed to the trauma itself rather than the surgical procedures. This suggested that trauma may directly impact B lymphocyte activity, potentially modulating the immune response (31).

The role of B lymphocytes in TMI has been less extensively studied. However, their immune regulatory functions suggest they may indirectly support repair processes by modulating macrophage activity or secreting specific cytokines. B lymphocytes can influence macrophage phenotypes by releasing cytokines such as IL-10 (211, 212), which may impact the healing trajectory following muscle injury. During the phases of skeletal muscle injury and repair, inflammatory cytokines like IFN-γ, IL-6 (213), and TNF-α (214) facilitate phagocytosis and play crucial roles in muscle repair. These cytokines, produced by various cell types, including B lymphocytes, contribute to the cytokine network and may coordinate muscle injury and repair processes.

The functions of B lymphocytes in muscle injury and repair may mirror their roles in other tissue injuries. In liver disease, B cell activity not only aids in controlling infections but may also exacerbate tissue damage and fibrosis by amplifying chronic inflammation (215). This suggests that B cells play a multifaceted role in tissue repair, promoting inflammatory responses and potentially hindering recovery by exacerbating inflammation. Moreover, disruptions in immune function following surgery or trauma may impair cell-mediated immunity, increasing susceptibility to infections. In the context of muscle injury, B lymphocytes may influence the repair process by modulating the immune response, regulating T cell activation and inhibition, and affecting the intensity and duration of inflammation (216, 217).

B lymphocytes contribute to TMI repair by directly participating in tissue repair through cytokine secretion and regulating inflammatory responses. These insights provide a valuable foundation for understanding the mechanisms by which B lymphocytes influence TMI repair.

## Therapeutic strategies targeting immune cells for TMI repair

In addition to conventional surgical management for advanced stages (Grade III and IV), current therapeutic strategies for TMI include the use of biomaterials, pharmacological agents, cell-based therapies, and physical rehabilitation approaches.

## Biomaterials effectively regulate the behavior of immune cells

As clearly illustrated in **Table 4**, biomaterial-based therapies for TMI improve the inflammatory microenvironment and support tissue regeneration by modulating immune cell activity. Cell delivery and tissue engineering strategies use cell carriers, such as stem cells, to establish biomimetic microenvironments, induce macrophage polarization for immune microenvironment optimization, and improve the healing in chronic injuries (220). Natural polymers include collagen and gelatin. They possess an inherent scaffold structure. This structure supports cell adhesion and proliferation (221). Natural polymers promote macrophage polarization. Specifically, they drive polarization from the pro-inflammatory M1 phenotype to the anti-inflammatory and reparative M2 phenotype. Additionally, they contribute to enhanced mechanical strength during muscle regeneration. They also aid in improving structural organization during muscle regeneration (218, 225). Immunomodulatory biomaterials, when combined with immunomodulators, modulate immune responses, attenuate inflammation, and immune rejection, and improve the immune microenvironment following muscle injury through the regulation of macrophage polarization (218). Bioprinted scaffolds offer precise three-dimensional structural support, facilitating cell distribution and proliferation, improving the local microenvironment, and modulating immune responses to enable *in situ* tissue regeneration and functional integration. Nanofiber scaffolds, characterized by their high specific surface area and porosity, provide attachment sites for cells, promote a favorable immune microenvironment, and induce macrophage polarization, improving the efficiency of tendon regeneration and enhancing mechanical properties (223). These therapies collectively target macrophages as central regulators, using the interplay between biomaterials and immune cells to resolve inflammation, support angiogenesis, and neurogenesis, and restore mechanical function following muscle injury. This represents a multi-technology-integrated immuno-regulatory biomaterial strategy for clinical tissue repair (224).

**Table 4:** Biomaterial-based therapies for TMI and immune cell involvement.

Classification Application	Biomaterial Technology	Specific Mechanism	Immune Cell Effect	Specific Efficacy	References
Acellular Scaffold Technology	Natural polymers (e.g., collagen and gelatin)	Exhibits excellent biocompatibility, biodegradability, and mechanical properties; alleviates inflammatory response; promotes stem cell proliferation and differentiation	Reduces inflammatory response; accelerates tissue regeneration	Mitigates post-tendon-injury inflammation and enhances regenerative capacity	(218)
Cell Delivery and Tissue Engineering	Cellular Carriers (e.g., Stem Cells)	Provides biomimetic microenvironment; facilitates cell migration and proliferation	Promotes macrophage polarization; improves immune microenvironment	Enhances healing quality and repair efficiency of chronic injuries	(219)
Bioactive Polymers and Electrical Stimulation	Bioactive Polymers (e.g., Hydrogels)	Modifies material surface properties; provides mechanical support and growth factor release	Promotes angiogenesis and neural precursor cell migration	Enhances tendon regenerative apacity and mechanical properties	(220)
Natural and Synthetic Materials	Natural Polymers (e.g., Collagen, Gelatin)	Provides native scaffold architecture; supports cell adhesion and proliferation	Promotes M1-to-M2 macrophage polarization	Improves mechanical properties and tissue architecture of tendon regeneration	(218, 221)
Mechanical Stimulation and Biomechanics	Dynamic Mechanical Stimulation Devices	Simulates biological environment; promotes tenocyte differentiation	Enhances cell adhesion and proliferation capacity	Improves mechanical properties and regenerative outcomes of tendon tissue	(222)
Immunomodulation	Biomaterials Combined with Immunomodulators	Regulates immune response; reduces inflammation and rejection	Promotes muscle tissue repair via macrophage polarization modulation	Improves post-muscle-injury immune microenvironment and accelerates tissue regeneration	(218)
Bioprinting Technology	Bioprinted Scaffolds	Provides precise three-dimensional structural support; facilitates cell distribution and proliferation	Improves local microenvironment; promotes immunomodulation	Achieves *in-situ* tissue regeneration and functional integration	(223)
Nanomaterials	Nanofibrous Scaffolds	Features high specific surface area and porosity; provides cellular adhesion sites	Improves immune microenvironment; promotes macrophage polarization	Enhances tendon regeneration efficiency and mechanical properties	(224)

## Drug intervention balances immune cells in the inflammatory response

Pharmacological interventions for TMI primarily aim to modulate inflammatory responses to preserve immune homeostasis and prevent excessive immune activation, which can hinder muscle regeneration (226). NSAIDs, such as aspirin, inhibit cyclooxygenase (COX) activity, reducing prostaglandin synthesis, directly suppressing neutrophil function, and effectively controlling acute inflammation, although concerns about their safety persist (227). β-adrenergic receptor antagonists promote the shift from Th1 to Th2 cell polarization, lowering the risk of post-traumatic infection and indirectly fostering a pro-regenerative immune microenvironment (228). Corticosteroids suppress the activity of various immune cells, including T cells (229) and macrophages (230), alleviating inflammation during the early injury phase and increasing cellular responsiveness to growth factors. This facilitates cell proliferation, migration, and differentiation, ultimately promoting muscle tissue repair (231).

## Cell therapy activates the regenerative potential of immune cells

Cellular therapies promote muscle regeneration by modulating specific immune cell populations. Tregs are key mediators in this process, secreting anti-inflammatory cytokines such as IL-10 and TGF-β to suppress pro-inflammatory signaling and establish a microenvironment conducive to tissue repair (232). Additionally, Tregs release IL-33, which directly stimulates myocyte regeneration, and facilitates the polarization of M1 macrophages toward the anti-inflammatory M2 phenotype, therefore reestablishing immune homeostasis (233). Treg infiltration peaks within four days post-injury, rapidly shifting the inflammatory landscape toward a reparative state (234). In parallel, stem cell therapies contribute to immune regulation by secreting immunomodulatory factors or replacing damaged immune cells, acting synergistically with other cell populations to support tissue regeneration (235).

## Physical intervention non-invasively regulates the function of immune cells

Physical interventions provide non-pharmacological strategies for immunomodulation in TMI. Massage therapy directly reduces neutrophil infiltration, disrupting inflammatory barriers that hinder muscle regeneration and exerting anti-inflammatory effects (136). Extremely low-frequency electromagnetic fields (ELF-EMF) modulate immune cell activity indirectly by regulating oxidative stress in muscle cells and increasing the expression of genes associated with myogenesis, establishing an immune microenvironment favorable for tissue repair (236). Both approaches support TMI recovery through non-invasive mechanisms.

In Summary, diverse immunotherapeutic strategies for TMI show translational promise across varying clinical readiness stages. Biomaterial-based therapies, such as decellularized scaffolds and hydrogels, modulate immune cell behavior to improve inflammatory microenvironments and support tissue regeneration, with preclinical validation demonstrating biocompatibility and efficacy, laying the groundwork for early-phase trials (237, 238). Pharmacological interventions, including NSAIDs and corticosteroids, are clinically used to regulate immune responses, though safety concerns (e.g., gastrointestinal risks with NSAIDs and delayed repair with long-term corticosteroid use) necessitate cautious dosing (239). Cell therapy can promote muscle regeneration by regulating the immune cell population. Particularly, Tregs secrete anti-inflammatory cytokines to establish a repair microenvironment, release IL-33 to stimulate muscle cell regeneration and M2-type phenotypic polarization (234). Although Tregs have been subject to clinical research in various diseases, their application in TMI is still in the stage of clinical research and early application (101). Physical interventions like massage therapy and ELF-EMF, which non-invasively regulate neutrophil infiltration and immune cell activity, are clinically established with favorable safety profiles (240). Emerging combined strategies (e.g., biomaterial-loaded CAR-Tregs, macrophage-targeted nanoparticles) represent promising preclinical approaches (241, 242). Ongoing trials aim to enhance specificity and safety by optimizing biomaterial scalability, refining cell delivery systems, and mitigating drug-related adverse effects. These advances collectively highlight the immune system’s fascinating yet complex role in TMI repair.

## Conclusions and future perspectives

Immune cells infiltrate the site of muscle damage. Following this infiltration, signaling molecules such as cytokines and growth factors are released into the microenvironment. These molecules regulate muscle repair and regeneration by directly interacting with satellite cells, as illustrated in **Figure 1**. Although previous studies have highlighted the crucial roles of neutrophils, macrophages, and Treg in skeletal muscle inflammation and tissue repair, significant gaps remain.

In recent years, the rapid development of single-cell (243) and spatially-resolved multiomics technologies has profoundly reshaping our understanding of the complexity of the immune response after traumatic injury (244). These technologies have revealed, with unprecedented resolution, the astonishing heterogeneity of immune cells during the injury repair process (245, 246). The trajectories of dynamic state transitions, and their precise localization and interaction relationships within the tissue space (247). For instance, single-cell transcriptomics has identified unique functional programs of macrophages and T cell subsets. These programs go beyond the traditional binary or simple classifications, such as M1/M2 for macrophages or Th1/Th2/Th17/Treg for T cells. Spatial multi-omics has illustrated how these immune subpopulations form specific spatial niches. These niches involve interactions with muscle stem cells, fibroblast/adipocyte progenitor cells, vascular cells, and other cell types (247). Through paracrine signals or direct contact, these immune subpopulations precisely regulate the regeneration process. Integrating these high-dimensional data with live dynamic imaging and computational biology is enabling systematic depiction. This integration is constructing a complete “cell map” and “interaction network” of the TMI immune microenvironment. Ultimately, it provides a powerful engine for deciphering the molecular logic underlying regeneration regulation (248).

Future research should focus on several critical aspects. The specific contributions and functional heterogeneity of immune cells in TMI repair remain unclear (249). By using single-cell transcriptomics and proteomics analysis, the subpopulation composition, activation status, and key secreted factors of these cells at different stages of injury can be systematically identified, and their unique roles in fibrosis regulation, angiogenesis, and extracellular matrix interactions can be clarified (250, 251). Targeted immunotherapy strategies may include modulating neutrophil-macrophage crosstalk to optimize inflammation-regeneration balance. This approach precisely regulates interactions between neutrophil subsets like N1/N2 and macrophage subpopulations using transformation patterns identified through single-cell analysis (252, 253). Another strategy involves genetic engineering of repair-promoting Treg subpopulations to overexpress immunomodulatory cytokines. Key Treg subsets or their signature molecules should be selected based on single-cell profiling (254, 255). Additionally, combining intelligent biomaterials with cell therapy can direct immune cell recruitment. Such biomaterials leverage tuned stiffness, topological features, and sustained-release capabilities to deliver specific ligands. These engineered systems recruit functionally validated pro-repair immune cells, including multiomics-identified macrophages or Tregs, to injury sites for enhanced regeneration (256, 257). In clinical translation, several promising approaches include: combined targeted therapy of immune cells and stem cells to treat refractory muscle injuries, implementing short-term anti-inflammatory intervention measures in sports medicine, and applying gene editing technology to enhance repair capabilities (257–259). Integrating systems biology with artificial intelligence to construct an immune map for muscle injury repair may help predict individualized treatment responses (256, 257).

Looking toward the future, the development of drug delivery systems with spatiotemporal specificity for fine-tuning the inflammation-regeneration balance, the investigation of immune crosstalk between organs, and the design of biomimetic materials that replicate the native extracellular matrix are imperative (13). Meanwhile, given the established close interaction between immune cells and traumatized muscles, the clinical translation of immunotherapy, based on current research findings, will offer a scientifically grounded approach to the management of injured muscles. A deeper understanding of immune-mediated repair mechanisms, combined with emerging technologies, will enable muscle regenerative medicine to move beyond symptom relief and achieve true functional recovery (260).

## Glossary

TMI: traumatic muscle injury
MRI: magnetic resonance imaging
IL: interleukin
TNF-α: tumor necrosis factor-α
TGF-β: transforming growth factor-β
CD8+ T cells: cluster of differentiation 8 positive T cells
IFN-γ: interferon-γ
MCP: monocyte chemoattractant protein
Tregs: regulatory T cells
M1: pro-inflammatory macrophage
M2: anti-inflammatory macrophage
CXCL: C-X-C motif chemokine ligand
NETs: neutrophil extracellular traps
ROS: reactive oxygen species
NAF: neutrophil-activating factor
HMGB1: high-mobility group box 1 protein
ADA: adenosine deaminase
CK: creatine kinase
MDA: malondialdehyde
NF-κB: nuclear factor kappa-light-chain-enhancer of activated B cells
MAPK: mitogen-activated protein kinase
STAT: signal transducer and activator of transcription
VEGF: vascular endothelial growth factor
NSAIDs: non-steroidal anti-inflammatory drugs
CAR-Tregs: chimeric antigen receptor-r T cells
EMBASE: excerpta medica database
GFEMs: glycoprotein 130-expressing macrophages
PPARγ/RXR: peroxisome proliferator-activated receptor gammaγ/retinoid X receptor
TKO: triple knockout
EGFR: epidermal growth factor receptor
GDF-15: growth differentiation factor
FAPs: fibro adipogenic progenitors
RIZs: regenerative inflammatory regions
ST: suppression of tumorigenicity
MMP-8: matrix metalloproteinase-8
SOD-2: superoxide dismutase 2
GPX: glutathione peroxidase
CAT: catalase
TRX: thioredoxin
TLRs: toll-like receptors
DAMPs: damage-associated molecular patterns
ERK: extracellular signal-regulated kinase
c-Jun N-terminal kinase: JNK
MPO: myeloperoxidase
PI3K: phosphoinositide 3-kinase
Akt: protein kinase B
mTOR: mammalian target of rapamycin
STAT1: signal transducer and activator of transcription 1
JAK1: janus kinase 1
NO: nitric oxide
PKC: protein kinase C
NAMPT: nicotinamide phosphoribosyl transferase
GM-CSF: granulocyte-macrophage colony-stimulating factor
IgE: immunoglobulin E
COX: cyclooxygenase
ELF-EMF: extremely low-frequency electromagnetic fields.

## References

[1] Edouard P Reurink G Mackey AL Lieber RL Pizzari T Järvinen TAH. Traumatic muscle injury. Nat Rev Dis Primers. (2023) 9:56. doi: 10.1038/s41572-023-00469-8, PMID: 37857686

[2] Oprescu SN Yue F Qiu J Brito LF Kuang S. Temporal dynamics and heterogeneity of cell populations during skeletal muscle regeneration. iScience. (2020) 23:100993. doi: 10.1016/j.isci.2020.100993, PMID: 32248062 PMC7125354

[3] Ekstrand J Hägglund M Waldén M. Epidemiology of muscle injuries in professional football (soccer). Am J Sports Med. (2011) 39:1226–32. doi: 10.1177/0363546510395879, PMID: 21335353

[4] Murphy MC Stannard J Sutton VR Owen PJ Park B Chivers PT. Epidemiology of musculoskeletal injury in military recruits: a systematic review and meta-analysis. BMC Sports Sci Med Rehabil. (2023) 15:144. doi: 10.1186/s13102-023-00755-8, PMID: 37898757 PMC10612319

[5] Briggs AM Woolf AD Dreinhöfer K Homb N Hoy DG Kopansky-Giles D. Reducing the global burden of musculoskeletal conditions. Bull World Health Organ. (2018) 96:366–8. doi: 10.2471/blt.17.204891, PMID: 29875522 PMC5985424

[6] Crowell MS Mason JS Morris JB Dummar MK Kuwik PA. Diagnostic imaging for distal extremity injuries in direct access physical therapy: an observational study. Int J Sports Phys Ther. (2023) 18:431–8. doi: 10.26603/001c.73314, PMID: 37020437 PMC10069368

[7] Dekker R Groothoff JW van der Sluis CK Eisma WH Ten Duis HJ. Long-term disabilities and handicaps following sports injuries: outcome after outpatient treatment. Disabil Rehabil. (2003) 25:1153–7. doi: 10.1080/0963828031000137757, PMID: 14534058

[8] Mueller-Wohlfahrt HW Haensel L Mithoefer K Ekstrand J English B McNally S. Terminology and classification of muscle injuries in sport: the Munich consensus statement. Br J Sports Med. (2013) 47:342–50. doi: 10.1136/bjsports-2012-091448, PMID: 23080315 PMC3607100

[9] Farrell SG Hatem M Bharam S. Acute adductor muscle injury: A systematic review on diagnostic imaging, treatment, and prevention. Am J Sports Med. (2023) 51:3591–603. doi: 10.1177/03635465221140923, PMID: 36661128

[10] Correction to "Fundamental principles of rehabilitation and musculoskeletal tissue healing. Vet Surg. (2025) 54:647. doi: 10.1111/vsu.14253, PMID: 40103197 PMC12128738

[11] Zhu J Fan J Xia Y Wang H Li Y Feng Z. Potential therapeutic targets of macrophages in inhibiting immune damage and fibrotic processes in musculoskeletal diseases. Front Immunol. (2023) 14:1219487. doi: 10.3389/fimmu.2023.1219487, PMID: 37545490 PMC10400722

[12] Qazi TH Duda GN Ort MJ Perka C Geissler S Winkler T. Cell therapy to improve regeneration of skeletal muscle injuries. J Cachexia Sarcopenia Muscle. (2019) 10:501–16. doi: 10.1002/jcsm.12416, PMID: 30843380 PMC6596399

[13] Carnes ME Pins GD. Skeletal muscle tissue engineering: biomaterials-based strategies for the treatment of volumetric muscle Loss. Bioengineering (Basel). (2020) 7:85. doi: 10.3390/bioengineering7030085, PMID: 32751847 PMC7552659

[14] Ye J Xie C Wang C Huang J Yin Z Heng BC. Promoting musculoskeletal system soft tissue regeneration by biomaterial-mediated modulation of macrophage polarization. Bioact Mater. (2021) 6:4096–109. doi: 10.1016/j.bioactmat.2021.04.017, PMID: 33997496 PMC8091177

[15] Feng P Che Y Gao C Chu X Li Z Li L. Profibrotic role of transcription factor SP1 in cross-talk between fibroblasts and M2 macrophages. iScience. (2023) 26:108484. doi: 10.1016/j.isci.2023.108484, PMID: 38094246 PMC10716550

[16] Kirkby Shaw K Alvarez L Foster SA Tomlinson JE Shaw AJ Pozzi A. Fundamental principles of rehabilitation and musculoskeletal tissue healing. Vet Surg. (2020) 49:22–32. doi: 10.1111/vsu.13270, PMID: 31271225 PMC6973127

[17] Costamagna D Berardi E Ceccarelli G Sampaolesi M. Adult stem cells and skeletal muscle regeneration. Curr Gene Ther. (2015) 15:348–63. doi: 10.2174/1566523215666150630121024, PMID: 26122100

[18] Hashimoto H Tamaki T Hirata M Uchiyama Y Sato M Mochida J. Reconstitution of the complete rupture in musculotendinous junction using skeletal muscle-derived multipotent stem cell sheet-pellets as a "bio-bond. PeerJ. (2016) 4:e2231. doi: 10.7717/peerj.2231, PMID: 27547541 PMC4957990

[19] McNamara SL Seo BR Freedman BR Roloson EB Alvarez JT O'Neill CT. Anti-inflammatory therapy enables robot-actuated regeneration of aged muscle. Sci Robot. (2023) 8:eadd9369. doi: 10.1126/scirobotics.add9369, PMID: 36947599 PMC10328427

[20] Skelton JK Purcell R. Preclinical models for studying immune responses to traumatic injury. Immunology. (2021) 162:377–88. doi: 10.1111/imm.13272, PMID: 32986856 PMC7968398

[21] Godoy-Tena G Barmada A Morante-Palacios O de la Calle-Fabregat C Martins-Ferreira R Ferreté-Bonastre AG. Epigenetic and transcriptomic reprogramming in monocytes of severe COVID-19 patients reflects alterations in myeloid differentiation and the influence of inflammatory cytokines. Genome Med. (2022) 14:134. doi: 10.1186/s13073-022-01137-4, PMID: 36443794 PMC9706884

[22] Klümper N Ralser DJ Bawden EG Landsberg J Zarbl R Kristiansen G. LAG3 (LAG-3, CD223) DNA methylation correlates with LAG3 expression by tumor and immune cells, immune cell infiltration, and overall survival in clear cell renal cell carcinoma. J Immunother Cancer. (2020) 8:e000552. doi: 10.1136/jitc-2020-000552, PMID: 32234847 PMC7174079

[23] Cillo AR Cardello C Shan F Karapetyan L Kunning S Sander C. Blockade of LAG-3 and PD-1 leads to co-expression of cytotoxic and exhaustion gene modules in CD8(+) T cells to promote antitumor immunity. Cell. (2024) 187:4373–4388.e4315. doi: 10.1016/j.cell.2024.06.036, PMID: 39121849 PMC11346583

[24] Spolski R Li P Chandra V Shin B Goel S Sakamoto K. Distinct use of super-enhancer elements controls cell type-specific CD25 transcription and function. Sci Immunol. (2023) 8:eadi8217. doi: 10.1126/sciimmunol.adi8217, PMID: 37922339 PMC10832512

[25] Seshadri A Brat GA Yorkgitis BK Keegan J Dolan J Salim A. Phenotyping the immune response to trauma: A multiparametric systems immunology approach. Crit Care Med. (2017) 45:1523–30. doi: 10.1097/ccm.0000000000002577, PMID: 28671900 PMC10114604

[26] Cayrol C Girard JP. Interleukin-33 (IL-33): A critical review of its biology and the mechanisms involved in its release as a potent extracellular cytokine. Cytokine. (2022) 156:155891. doi: 10.1016/j.cyto.2022.155891, PMID: 35640416

[27] Fukuda K Ishida W Kishimoto T Nakajima I Miura Y Sumi T. Role of damage-associated molecular patterns (DAMPs/alarmins) in severe ocular allergic diseases. Cells. (2022) 11:1051. doi: 10.3390/cells11061051, PMID: 35326502 PMC8946931

[28] Land WG Agostinis P Gasser S Garg AD Linkermann A. Transplantation and damage-associated molecular patterns (DAMPs). Am J Transplant. (2016) 16:3338–61. doi: 10.1111/ajt.13963, PMID: 27421829

[29] Li L Lu YQ. The regulatory role of high-mobility group protein 1 in sepsis-related immunity. Front Immunol. (2020) 11:601815. doi: 10.3389/fimmu.2020.601815, PMID: 33552058 PMC7862754

[30] Tian T Lofftus S Pan Y Stingley CA King SL Zhao J. IL1α antagonizes IL1β and promotes adaptive immune rejection of Malignant tumors. Cancer Immunol Res. (2020) 8:660–71. doi: 10.1158/2326-6066.Cir-19-0552, PMID: 32161110 PMC7596693

[31] Chakraborty S Karasu E Huber-Lang M. Complement after trauma: Suturing innate and adaptive immunity. Front Immunol. (2018) 9:2050. doi: 10.3389/fimmu.2018.02050, PMID: 30319602 PMC6165897

[32] Cole E Gillespie S Vulliamy P Brohi K. Multiple organ dysfunction after trauma. Br J Surg. (2020) 107:402–12. doi: 10.1002/bjs.11361, PMID: 31691956 PMC7078999

[33] Li R Ye JJ Gan L Zhang M Sun D Li Y. Traumatic inflammatory response: pathophysiological role and clinical value of cytokines. Eur J Trauma Emerg Surg. (2024) 50:1313–30. doi: 10.1007/s00068-023-02388-5, PMID: 38151578 PMC11458723

[34] Ma M Jiang W Zhou R. DAMPs and DAMP-sensing receptors in inflammation and diseases. Immunity. (2024) 57:752–71. doi: 10.1016/j.immuni.2024.03.002, PMID: 38599169

[35] Place DE Kanneganti TD. The innate immune system and cell death in autoinflammatory and autoimmune disease. Curr Opin Immunol. (2020) 67:95–105. doi: 10.1016/j.coi.2020.10.013, PMID: 33242752

[36] Panicucci C Raffaghello L Bruzzone S Baratto S Principi E Minetti C. eATP/P2X7R Axis: An orchestrated pathway triggering inflammasome activation in muscle diseases. Int J Mol Sci. (2020) 21:5963. doi: 10.3390/ijms21175963, PMID: 32825102 PMC7504480

[37] Arnold L Henry A Poron F Baba-Amer Y van Rooijen N Plonquet A. Inflammatory monocytes recruited after skeletal muscle injury switch into antiinflammatory macrophages to support myogenesis. J Exp Med. (2007) 204:1057–69. doi: 10.1084/jem.20070075, PMID: 17485518 PMC2118577

[38] Yan L Wang J Cai X Liou YC Shen HM Hao J. Macrophage plasticity: signaling pathways, tissue repair, and regeneration. MedComm. (2020) e658. doi: 10.1002/mco2.658, PMID: 39092292 PMC11292402

[39] Hu W Shi J Lv W Jia X Ariga K. Regulation of stem cell fate and function by using bioactive materials with nanoarchitectonics for regenerative medicine. Sci Technol Adv Mater. (2022) 23:393–412. doi: 10.1080/14686996.2022.2082260, PMID: 35783540 PMC9246028

[40] Liu J Xiao Q Xiao J Niu C Li Y Zhang X. Wnt/β-catenin signalling: function, biological mechanisms, and therapeutic opportunities. Signal Transduct Target Ther. (2022) 7:3. doi: 10.1038/s41392-021-00762-6, PMID: 34980884 PMC8724284

[41] Larouche JA Fraczek PM Kurpiers SJ Yang BA Davis C Castor-Macias JA. Neutrophil and natural killer cell imbalances prevent muscle stem cell-mediated regeneration following murine volumetric muscle loss. Proc Natl Acad Sci U.S.A. (2022) 119:e2111445119. doi: 10.1073/pnas.2111445119, PMID: 35377804 PMC9169656

[42] Arjomandnejad M Kopec AL Keeler AM. CAR-T regulatory (CAR-treg) cells: engineering and applications. Biomedicines. (2022) 10:287. doi: 10.3390/biomedicines10020287, PMID: 35203496 PMC8869296

[43] Verhagen E Clarsen B van der Graaff L Bahr R. Do not neglect injury severity and burden when assessing the effect of sports injury prevention interventions: time to paint the whole picture. Br J Sports Med. (2024) 58:1166–9. doi: 10.1136/bjsports-2024-108215, PMID: 38969484 PMC11503035

[44] Flores DV Mejía Gómez C Estrada-Castrillón M Smitaman E Pathria MN. MR Imaging of muscle trauma: Anatomy, biomechanics, pathophysiology, and imaging appearance. Radiographics. (2018) 38:124–48. doi: 10.1148/rg.2018170072, PMID: 29220207

[45] Duque J Bonfanti A Fouchard J Baldauf L Azenha SR Ferber E. Rupture strength of living cell monolayers. Nat Mater. (2024) 23:1563–74. doi: 10.1038/s41563-024-02027-3, PMID: 39468334 PMC11525174

[46] Fang XX Zhai MN Zhu M He C Wang H Wang J. Inflammation in pathogenesis of chronic pain: Foe and friend. Mol Pain. (2023) 19:17448069231178176. doi: 10.1177/17448069231178176, PMID: 37220667 PMC10214073

[47] Markus I Constantini K Hoffman JR Bartolomei S Gepner Y. Exercise-induced muscle damage: mechanism, assessment and nutritional factors to accelerate recovery. Eur J Appl Physiol. (2021) 121:969–92. doi: 10.1007/s00421-020-04566-4, PMID: 33420603

[48] Dabbagh A MacDermid JC. Appraisal of clinical practice guideline: Clinical practice guidelines for pain management in acute musculoskeletal Injury. J Physiother. (2020) 66:134. doi: 10.1016/j.jphys.2020.02.006, PMID: 32291215

[49] Hossain M Kubes P. Innate immune cells orchestrate the repair of sterile injury in the liver and beyond. Eur J Immunol. (2019) 49:831–41. doi: 10.1002/eji.201847485, PMID: 31001813

[50] Fernandez A Aubry-Rozier B Vautey M Berna C Suter MR. Small fiber neuropathy in hypermobile Ehlers Danlos syndrome/hypermobility spectrum disorder. J Intern Med. (2022) 292:957–60. doi: 10.1111/joim.13539, PMID: 35781355 PMC9796626

[51] Mizumura K Taguchi T. Neurochemical mechanism of muscular pain: Insight from the study on delayed onset muscle soreness. J Physiol Sci. (2024) 74:4. doi: 10.1186/s12576-023-00896-y, PMID: 38267849 PMC10809664

[52] Sastourné-Arrey Q Mathieu M Contreras X Monferran S Bourlier V Gil-Ortega M. Adipose tissue is a source of regenerative cells that augment the repair of skeletal muscle after injury. Nat Commun. (2023) 14:80. doi: 10.1038/s41467-022-35524-7, PMID: 36604419 PMC9816314

[53] Hotfiel T Freiwald J Hoppe MW Lutter C Forst R Grim C. Advances in delayed-onset muscle soreness (DOMS): part I: pathogenesis and diagnostics. Sportverletz SportsChaden. (2018) 32:243–50. doi: 10.1055/a-0753-1884, PMID: 30537791

[54] Hou J Nelson R Mohammad N Mustafa G Plant D Thompson FJ. Effect of simultaneous combined treadmill training and magnetic stimulation on spasticity and gait impairments after cervical spinal cord injury. J Neurotrauma. (2020) 37:1999–2013. doi: 10.1089/neu.2019.6961, PMID: 32340533

[55] Metsemakers WJ Onsea J Moriarty TF Pruidze N Nadareishvili L Dadiani M. Bacteriophage therapy for human musculoskeletal and skin/soft tissue infections. Clin Microbiol Infect. (2023) 29:695–701. doi: 10.1016/j.cmi.2023.01.011, PMID: 36669559

[56] Peake JM Neubauer O Della Gatta PA Nosaka K. Muscle damage and inflammation during recovery from exercise. J Appl Physiol (1985). (2017) 122:559–70. doi: 10.1152/japplphysiol.00971.2016, PMID: 28035017

[57] Venance SL. Approach to the patient with hyperCKemia. Continuum (Minneap Minn). (2016) 22:1803–14. doi: 10.1212/01.Con.0000511069.68640.39, PMID: 27922494

[58] van de Velde NM Koeks Z Signorelli M Verwey N Overzier M Bakker JA. Longitudinal assessment of creatine kinase, creatine/creatinine(ratio), and myostatin as monitoring biomarkers in decker muscular dystrophy. Neurology. (2023) 100:e975–84. doi: 10.1212/wnl.0000000000201609, PMID: 36849458 PMC9990441

[59] Granella LL Chielle EO Maziero JS Vidigal TMA Mallmanna BLK Karal J. Evaluating muscle damage biomarkers in adolescent athletes: implications for public health in tacna, Peru-2023. Int J Environ Res Public Health. (2024) 21:1394. doi: 10.3390/ijerph21111394, PMID: 39595661 PMC11593879

[60] Wu H Zhu H Yuan C Yao C Luo W Shen X. Clinical and immune features of hospitalized pediatric patients with coronavirus disease 2019 (COVID-19) in Wuhan, China. JAMA Netw Open. (2020) 3:e2010895. doi: 10.1001/jamanetworkopen.2020.10895, PMID: 32492165 PMC7272117

[61] Ho TH Lin LC Tseng WC Tsai CK. The index of physical performance in muscle damage biomarker in youth athletes: 2359 Board 278 May 28 3:00 PM - 4:30 PM. Med Sci Sports Exercise. (2020) 52:693. doi: 10.1249/01.mss.0000681228.36820.0

[62] Aggio V Fabbella L Poletti S Lorenzi C Finardi A Colombo C. Circulating cytotoxic immune cell composition, activation status and toxins expression associate with white matter microstructure in bipolar disorder. Sci Rep. (2023) 13:22209. doi: 10.1038/s41598-023-49146-6, PMID: 38097657 PMC10721611

[63] Rouget C Girardot T Textoris J Monneret G Rimmelé T Venet F. Biological markers of injury-induced immunosuppression. Minerva Anestesiol. (2017) 83:302–314. doi: 10.23736/s0375-9393.16.11268-4, PMID: 27314598

[64] Lerchbaumer MH Perschk M Gwinner C. Ultrasound in sports traumatology. Rofo. (2024) 196:440–9. doi: 10.1055/a-2185-8264, PMID: 37944936

[65] Kuo FH Baumann HM D'Empaire PP Deng Y. Role of point-of-care ultrasound in the early stages of trauma care. Curr Anesthesiology Rep. (2020) 10:1–11. doi: 10.1002/ijc.31357, PMID: 29516506

[66] Abd EMM Hatem SM Yehia AE. Evaluation of tendoAchilles lesions by (MRI). QJM: Int J Med. (2021) Supplement_1. doi: 10.1093/qjmed/hcab106.043

[67] Du MJ Lin YH Chen WT Zhao H. Advances in the application of ultrasound for fracture diagnosis and treatment. Eur Rev Med Pharmacol Sci. (2022) 26:7949–54. doi: 10.26355/eurrev_202211_30146, PMID: 36394743

[68] Elliott JM Smith AC Hoggarth MA Albin SR Weber KA 2nd Haager M. Muscle fat infiltration following whiplash: A computed tomography and magnetic resonance imaging comparison. PloS One. (2020) 15:e0234061. doi: 10.1371/journal.pone.0234061, PMID: 32484835 PMC7266316

[69] Bischof K Stafilidis S Bundschuh L Oesser S Baca A König D. Influence of specific collagen peptides and 12-week concurrent training on recovery-related biomechanical characteristics following exercise-induced muscle damage-A randomized controlled trial. Front Nutr. (2023) 10:1266056. doi: 10.3389/fnut.2023.1266056, PMID: 38035363 PMC10687431

[70] Bindu S Mazumder S Bandyopadhyay U. Non-steroidal anti-inflammatory drugs (NSAIDs) and organ damage: A current perspective. Biochem Pharmacol. (2020) 180:114147. doi: 10.1016/j.bcp.2020.114147, PMID: 32653589 PMC7347500

[71] Day RO Graham GG. Non-steroidal anti-inflammatory drugs (NSAIDs). BMJ. (2013) 346:f3195. doi: 10.1136/bmj.f3195, PMID: 23757736

[72] Muscatelli SR Charters MA Hallstrom BR. Time for an Update? A look at current guidelines for venous thromboembolism prophylaxis after hip and knee arthroplasty and hip fracture. Arthroplast Today. (2021) 10:105–7. doi: 10.1016/j.artd.2021.06.015, PMID: 34337116 PMC8318891

[73] Guermazi A Roemer FW Robinson P Tol JL Regatte RR Crema MD. Imaging of muscle injuries in sports medicine: Sports imaging series. Radiology. (2017) 282:646–63. doi: 10.1148/radiol.2017160267, PMID: 28218878

[74] Zadro JR O'Keeffe M Ferreira GE Haas R Harris IA Buchbinder R. Diagnostic labels for rotator cuff disease can increase people's perceived need for shoulder surgery: An online randomized controlled trial. J Orthop Sports Phys Ther. (2021) 51:401–11. doi: 10.2519/jospt.2021.10375, PMID: 33789444

[75] Elmashala A Rosand J. Management of subdural haematoma: optimising drainage. Lancet Neurol. (2024) 23:751–2. doi: 10.1016/s1474-4422(24)00218-7, PMID: 38878791

[76] Yaseen W Kraft-Sheleg O Zaffryar-Eilot S Melamed S Sun C Millay DP. Fibroblast fusion to the muscle fiber regulates myotendinous junction formation. Nat Commun. (2021) 12:3852. doi: 10.1038/s41467-021-24159-9, PMID: 34158500 PMC8219707

[77] Lee SY Park J. Emotional changes and functional progressions during post-operative rehabilitation in collegiate student-athletes: A preliminary study. Healthcare (Basel). (2021) 9:184. doi: 10.3390/healthcare9020184, PMID: 33572279 PMC7916025

[78] Della Villa F Massa B Bortolami A Nanni G Olmo J Virgile A. Infographic. Injury mechanisms and situational patterns of severe lower limb muscle injuries in male professional football (soccer) players: a systematic video analysis study on 103 cases. Br J Sports Med. (2024) 58:689–90. doi: 10.1136/bjsports-2023-107908, PMID: 38527769

[79] Kumaravel M Bawa P Murai N. Magnetic resonance imaging of muscle injury in elite American football players: Predictors for return to play and performance. Eur J Radiol. (2018) 108:155–64. doi: 10.1016/j.ejrad.2018.09.028, PMID: 30396649

[80] Yang W Hu P. Skeletal muscle regeneration is modulated by inflammation. J Orthop Translat. (2018) 13:25–32. doi: 10.1016/j.jot.2018.01.002, PMID: 29662788 PMC5892385

[81] San Emeterio CL Olingy CE Chu Y Botchwey EA. Selective recruitment of non-classical monocytes promotes skeletal muscle repair. Biomaterials. (2017) 117:32–43. doi: 10.1016/j.biomaterials.2016.11.021, PMID: 27930948 PMC5218730

[82] Fu X Zhuang CL Hu P. Regulation of muscle stem cell fate. Cell Regen. (2022) 11:40. doi: 10.1186/s13619-022-00142-7, PMID: 36456659 PMC9715903

[83] Qi B Li Y Peng Z Luo Z Zhang X Chen J. Macrophage-myofibroblast transition as a potential origin for skeletal muscle fibrosis after injury via complement system activation. J Inflammation Res. (2024) 17:1083–94. doi: 10.2147/jir.S450599, PMID: 38384372 PMC10880461

[84] Gorga SM Carlton E Kohne J Barbaro R. 1432: Kidney injury incidence in multiple organ dysfunction syndrome. Crit Care Med. (2020) 84:693. doi: 10.1097/01.ccm.0000645644.62852.9b

[85] Zhao H Wang Y Shao Y Liu J Wang S Xing M. Oxidative stress-induced skeletal muscle injury involves in NF-κB/p53-activated immunosuppression and apoptosis response in copper (II) or/and arsenite-exposed chicken. Chemosphere. (2018) 210:76–84. doi: 10.1016/j.chemosphere.2018.06.165, PMID: 29986226

[86] Hazeldine J Naumann DN Toman E Davies D Bishop JRB Su Z. Prehospital immune responses and development of multiple organ dysfunction syndrome following traumatic injury: A prospective cohort study. PloS Med. (2017) 14:e1002338. doi: 10.1371/journal.pmed.1002338, PMID: 28719602 PMC5515405

[87] Sun L Wang X Saredy J Yuan Z Yang X Wang H. Innate-adaptive immunity interplay and redox regulation in immune response. Redox Biol. (2020) 37:101759. doi: 10.1016/j.redox.2020.101759, PMID: 33086106 PMC7575795

[88] Jarczak D Nierhaus A. Cytokine storm-definition, causes, and implications. Int J Mol Sci. (2022) 23:11740. doi: 10.3390/ijms231911740, PMID: 36233040 PMC9570384

[89] Pape HC Moore EE McKinley T Sauaia A. Pathophysiology in patients with polytrauma. Injury. (2022) 53:2400–12. doi: 10.1016/j.injury.2022.04.009, PMID: 35577600

[90] Sun Q Chen E. Significance of heparin in the treatment of patients with sepsis. Zhonghua Wei Zhong Bing Ji Jiu Yi Xue. (2017) 29:1144–7. doi: 10.3760/cma.j.issn.2095-4352.2017.12.019, PMID: 29216953

[91] Ziemkiewicz N Hilliard G Pullen NA Garg K. The role of innate and adaptive immune cells in skeletal muscle regeneration. Int J Mol Sci. (2021) 22:3265. doi: 10.3390/ijms22063265, PMID: 33806895 PMC8005179

[92] Yan D Yu F Chen L Yao Q Yan C Zhang S. Subconjunctival injection of regulatory T cells potentiates corneal healing via orchestrating inflammation and tissue repair after acute alkali burn. Invest Ophthalmol Vis Sci. (2020) 61:22. doi: 10.1167/iovs.61.14.22, PMID: 33326018 PMC7745601

[93] Edderkaoui B. Chemokines in cartilage regeneration and degradation: New Insights. Int J Mol Sci. (2023) 25:381. doi: 10.3390/ijms25010381, PMID: 38203552 PMC10779035

[94] Ferreira LB Williams KA Best G Haydinger CD Smith JR. Inflammatory cytokines as mediators of retinal endothelial barrier dysfunction in non-infectious uveitis. Clin Transl Immunol. (2023) 12:e1479. doi: 10.1002/cti2.1479, PMID: 38090668 PMC10714664

[95] Xie Z Zhang M Zhou G Lin L Han J Wang Y. Emerging roles of the Hedgehog signalling pathway in inflammatory bowel disease. Cell Death Discov. (2021) 7:314. doi: 10.1038/s41420-021-00679-7, PMID: 34702800 PMC8548344

[96] Han AA Currie HN Loos MS Scardoni G Miller JV Prince N. The impact of cytokine responses in the intra- and extracellular signaling network of a traumatic injury. Cytokine. (2018) 106:136–47. doi: 10.1016/j.cyto.2017.10.027, PMID: 29103821 PMC5913004

[97] Peiseler M Kubes P. More friend than foe: the emerging role of neutrophils in tissue repair. J Clin Invest. (2019) 129:2629–39. doi: 10.1172/jci124616, PMID: 31205028 PMC6597202

[98] Yan W Li T Yin T Hou Z Qu K Wang N. M2 macrophage-derived exosomes promote the c-KIT phenotype of vascular smooth muscle cells during vascular tissue repair after intravascular stent implantation. Theranostics. (2020) 10:10712–28. doi: 10.7150/thno.46143, PMID: 32929376 PMC7482821

[99] Tang Q Dong C Sun Q. Immune response associated with ischemia and reperfusion injury during organ transplantation. Inflammation Res. (2022) 71:1463–76. doi: 10.1007/s00011-022-01651-6, PMID: 36282292 PMC9653341

[100] Schiaffino S Pereira MG Ciciliot S Rovere-Querini P. Regulatory T cells and skeletal muscle regeneration. FEBS J. (2017) 284:517–24. doi: 10.1111/febs.13827, PMID: 27479876

[101] Wu J Ren B Wang D Lin H. Regulatory T cells in skeletal muscle repair and regeneration: recent insights. Cell Death Dis. (2022) 13:680. doi: 10.1038/s41419-022-05142-8, PMID: 35931697 PMC9356005

[102] Kawashima M Miyakawa M Sugiyama M Miyoshi M Arakawa T. Unloading during skeletal muscle regeneration retards iNOS-expressing macrophage recruitment and perturbs satellite cell accumulation. Histochem Cell Biol. (2020) 154:355–67. doi: 10.1007/s00418-020-01897-3, PMID: 32617655

[103] Maier C Ramming A Bergmann C Weinkam R Kittan N Schett G. Inhibition of phosphodiesterase 4 (PDE4) reduces dermal fibrosis by interfering with the release of interleukin-6 from M2 macrophages. Ann Rheum Dis. (2017) 76:1133–41. doi: 10.1136/annrheumdis-2016-210189, PMID: 28209630

[104] Shu LZ Zhang XL Ding YD Lin H. From inflammation to bone formation: the intricate role of neutrophils in skeletal muscle injury and traumatic heterotopic ossification. Exp Mol Med. (2024) 56:1523–30. doi: 10.1038/s12276-024-01270-7, PMID: 38945957 PMC11297321

[105] Khalaf NB Al-Mehatab D Fathallah DM. Vascular endothelial ERp72 is involved in the inflammatory response in a rat model of skeletal muscle injury. Mol Med Rep. (2021) 23:186. doi: 10.3892/mmr.2021.11825, PMID: 33398381 PMC7809907

[106] Grudzinska FS Sapey E. Friend or foe? The dual role of neutrophils in lung injury and repair. Thorax. (2018) 73:305–7. doi: 10.1136/thoraxjnl-2017-211253, PMID: 29382796

[107] Wang J. Neutrophils in tissue injury and repair. Cell Tissue Res. (2018) 371:531–9. doi: 10.1007/s00441-017-2785-7, PMID: 29383445 PMC5820392

[108] Manole CG Voiculescu VM Soare C Ceafalan LC Gherghiceanu M Hinescu ME. Skin telocytes could fundament the cellular mechanisms of wound healing in platelet-rich plasma Administration. Cells. (2024) 13:1321. doi: 10.3390/cells13161321, PMID: 39195210 PMC11353115

[109] Westman J Grinstein S Marques PE. Phagocytosis of necrotic debris at sites of injury and inflammation. Front Immunol. (2019) 10:3030. doi: 10.3389/fimmu.2019.03030, PMID: 31998312 PMC6962235

[110] Barkaway A Rolas L Joulia R Bodkin J Lenn T Owen-Woods C. Age-related changes in the local milieu of inflamed tissues cause aberrant neutrophil trafficking and subsequent remote organ damage. Immunity. (2021) 54:1494–1510.e1497. doi: 10.1016/j.immuni.2021.04.025, PMID: 34033752 PMC8284598

[111] Schnoor M Alcaide P Voisin MB van Buul JD. Crossing the Vascular Wall: Crossing the vascular wall: cmmon and unique mechanisms exploited by different leukocyte subsets during extravasation. Mediators Inflamm. (2015) 2015:946509. doi: 10.1155/2015/946509, PMID: 26568666 PMC4629053

[112] Lippitz BE Harris RA. Cytokine patterns in cancer patients: A review of the correlation between interleukin 6 and prognosis. Oncoimmunology. (2016) 5:e1093722. doi: 10.1080/2162402x.2015.1093722, PMID: 27467926 PMC4910721

[113] Tecchio C Micheletti A Cassatella MA. Neutrophil-derived cytokines: facts beyond expression. Front Immunol. (2014) 5:508. doi: 10.3389/fimmu.2014.00508, PMID: 25374568 PMC4204637

[114] Castanheira FVS Kubes P. Neutrophils and NETs in modulating acute and chronic inflammation. Blood. (2019) 133:2178–85. doi: 10.1182/blood-2018-11-844530, PMID: 30898862

[115] Torres-Ruiz J Alcalá-Carmona B Alejandre-Aguilar R Gómez-Martín D. Inflammatory myopathies and beyond: The dual role of neutrophils in muscle damage and regeneration. Front Immunol. (2023) 14:1113214. doi: 10.3389/fimmu.2023.1113214, PMID: 36923415 PMC10008923

[116] Howard EE Pasiakos SM Blesso CN Fussell MA Rodriguez NR. Divergent roles of inflammation in skeletal muscle recovery from injury. Front Physiol. (2020) 11:87. doi: 10.3389/fphys.2020.00087, PMID: 32116792 PMC7031348

[117] Papayannopoulos V. Neutrophil extracellular traps in immunity and disease. Nat Rev Immunol. (2018) 18:134–47. doi: 10.1038/nri.2017.105, PMID: 28990587

[118] Leliefeld PH Wessels CM Leenen LP Koenderman L Pillay J. The role of neutrophils in immune dysfunction during severe inflammation. Crit Care. (2016) 20:73. doi: 10.1186/s13054-016-1250-4, PMID: 27005275 PMC4804478

[119] Ohms M Möller S Laskay T. An attempt to polarize human neutrophils toward N1 and N2 phenotypes *in vitro* . Front Immunol. (2020) 11:532. doi: 10.3389/fimmu.2020.00532, PMID: 32411122 PMC7198726

[120] Daseke MJ 2nd Chalise U Becirovic-Agic M Salomon JD Cook LM Case AJ. Neutrophil signaling during myocardial infarction wound repair. Cell Signal. (2021) 77:109816. doi: 10.1016/j.cellsig.2020.109816, PMID: 33122000 PMC7718402

[121] Prame Kumar K Nicholls AJ Wong CHY. Partners in crime: neutrophils and monocytes/macrophages in inflammation and disease. Cell Tissue Res. (2018) 371:551–65. doi: 10.1007/s00441-017-2753-2, PMID: 29387942 PMC5820413

[122] Cuartero MI Ballesteros I Moraga A Nombela F Vivancos J Hamilton JA. N2 neutrophils, novel players in brain inflammation after stroke: modulation by the PPARγ agonist rosiglitazone. Stroke. (2013) 44:3498–508. doi: 10.1161/strokeaha.113.002470, PMID: 24135932

[123] Yang L Shi F Cao F Wang L She J He B. Neutrophils in tissue injury and repair: Molecular mechanisms and therapeutic yargets. MedComm. (2020) e70184. doi: 10.1002/mco2.70184, PMID: 40260014 PMC12010766

[124] Fuertes-Alvarez S Izeta A. Terminal schwann cell aging: implications for age-associated neuromuscular dysfunction. Aging Dis. (2021) 12:494–514. doi: 10.14336/ad.2020.0708, PMID: 33815879 PMC7990373

[125] Walter LD Orton JL Ntekas I Fong EHH Maymi VI Rudd BD. Transcriptomic analysis of skeletal muscle regeneration across mouse lifespan identifies altered stem cell states. Nat Aging. (2024) 4:1862–81. doi: 10.1038/s43587-024-00756-3, PMID: 39578558 PMC11645289

[126] Chen M Pan Y Liu H Ning F Lu Q Duan Y. Ezrin accelerates breast cancer liver metastasis through promoting furin-like convertase-mediated cleavage of Notch1. Cell Oncol (Dordr). (2023) 46:571–87. doi: 10.1007/s13402-022-00761-x, PMID: 36580262 PMC12974704

[127] Ip WKE Hoshi N Shouval DS Snapper S Medzhitov R. Anti-inflammatory effect of IL-10 mediated by metabolic reprogramming of macrophages. Science. (2017) 356:513–9. doi: 10.1126/science.aal3535, PMID: 28473584 PMC6260791

[128] Pratt HG Steinberger KJ Mihalik NE Ott S Whalley T Szomolay B. Macrophage and neutrophil interactions in the pancreatic tumor microenvironment drive the pathogenesis of pancreatic cancer. Cancers (Basel). (2021) 14:194. doi: 10.3390/cancers14010194, PMID: 35008355 PMC8750413

[129] Giebel B. On the function and heterogeneity of extracellular vesicles. Ann Trans Med. (2017) 5:150. doi: 10.21037/atm.2017.02.14, PMID: 28462230 PMC5395490

[130] Egan B Sharples AP. Molecular responses to acute exercise and their relevance for adaptations in skeletal muscle to exercise training. Physiol Rev. (2023) 103:2057–170. doi: 10.1152/physrev.00054.2021, PMID: 36395350

[131] Smith C Kruger MJ Smith RM Myburgh KH. The inflammatory response to skeletal muscle injury: illuminating complexities. Sports Med. (2008) 38:947–69. doi: 10.2165/00007256-200838110-00005, PMID: 18937524

[132] Ouyang N Zhao Y Chen Q Chen L Fang B Dai J. The effect of celecoxib in traumatic heterotopic ossification around temporomandibular joint in mice. Osteoarthritis Cartilage. (2020) 28:502–15. doi: 10.1016/j.joca.2020.01.014, PMID: 32061965

[133] Babatunde KA Datta R Hendrikse NW Ayuso JM Huttenlocher A Skala MC. Naive primary neutrophils play a dual role in the tumor microenvironment. iScience. (2024) 27:110632. doi: 10.1016/j.isci.2024.110632, PMID: 39246449 PMC11379674

[134] Tulangekar A Sztal TE. Inflammation in duchenne muscular dystrophy-exploring the role of neutrophils in muscle damage and regeneration. Biomedicines. (2021) 9. doi: 10.3390/biomedicines9101366, PMID: 34680483 PMC8533596

[135] Seo BR Payne CJ McNamara SL Freedman BR Kwee BJ Nam S. Skeletal muscle regeneration with robotic actuation-mediated clearance of neutrophils. Sci Transl Med. (2021) 13:eabe8868. doi: 10.1126/scitranslmed.abe8868, PMID: 34613813 PMC8961724

[136] He Y Heng Y Qin Z Wei X Wu Z Qu J. Intravital microscopy of satellite cell dynamics and their interaction with myeloid cells during skeletal muscle regeneration. Sci Adv. (2023) 9:eadi1891. doi: 10.1126/sciadv.adi1891, PMID: 37851799 PMC10584350

[137] Forcina L Miano C Pelosi L Musarò A. An overview about the biology of skeletal muscle satellite cells. Curr Genomics. (2019) 20:24–37. doi: 10.2174/1389202920666190116094736, PMID: 31015789 PMC6446479

[138] Lu YZ Nayer B Singh SK Alshoubaki YK Yuan E Park AJ. CGRP sensory neurons promote tissue healing via neutrophils and macrophages. Nature. (2024) 628:604–11. doi: 10.1038/s41586-024-07237-y, PMID: 38538784 PMC11023938

[139] Ding H Chen S Pan X Dai X Pan G Li Z. Transferrin receptor 1 ablation in satellite cells impedes skeletal muscle regeneration through activation of ferroptosis. J Cachexia Sarcopenia Muscle. (2021) 12:746–68. doi: 10.1002/jcsm.12700, PMID: 33955709 PMC8200440

[140] Leppkes M Lindemann A Gößwein S Paulus S Roth D Hartung A. Neutrophils prevent rectal bleeding in ulcerative colitis by peptidyl-arginine deiminase-4-dependent immunothrombosis. Gut. (2022) 71:2414–29. doi: 10.1136/gutjnl-2021-324725, PMID: 34862250 PMC9667856

[141] Loh W Vermeren S. Anti-inflammatory neutrophil functions in the resolution of inflammation and tissue repair. Cells. (2022) 11:4076. doi: 10.3390/cells11244076, PMID: 36552840 PMC9776979

[142] Teixeira CFP Zamunér SR Zuliani JP Fernandes CM Cruz-Hofling MA Fernandes I. Neutrophils do not contribute to local tissue damage, but play a key role in skeletal muscle regeneration, in mice injected with Bothrops asper snake venom. Muscle Nerve. (2003) 28:449–59. doi: 10.1002/mus.10453, PMID: 14506717

[143] Dumont N Bouchard P Frenette J. Neutrophil-induced skeletal muscle damage: a calculated and controlled response following hindlimb unloading and reloading. Am J Physiol Regul Integr Comp Physiol. (2008) 295:R1831–1838. doi: 10.1152/ajpregu.90318.2008, PMID: 18784335

[144] Lokwani R Josyula A Ngo TB DeStefano S Fertil D Faust M. Pro-regenerative biomaterials recruit immunoregulatory dendritic cells after traumatic injury. Nat Mater. (2024) 23:147–57. doi: 10.1038/s41563-023-01689-9, PMID: 37872423 PMC13504775

[145] Aratani Y. Myeloperoxidase: Its role for host defense, inflammation, and neutrophil function. Arch Biochem Biophys. (2018) 640:47–52. doi: 10.1016/j.abb.2018.01.004, PMID: 29336940

[146] Chung TH Hsieh CC Hsiao JK Hsu SC Yao M Huang DM. Dextran-coated iron oxide nanoparticles turn protumor mesenchymal stem cells (MSCs) into antitumor MSCs. Rsc Adv. (2016) 6:45553–61. doi: 10.1039/C6RA03453E

[147] Kozakowska M Pietraszek-Gremplewicz K Jozkowicz A Dulak J. The role of oxidative stress in skeletal muscle injury and regeneration: focus on antioxidant enzymes. J Muscle Res Cell Motil. (2015) 36:377–93. doi: 10.1007/s10974-015-9438-9, PMID: 26728750 PMC4762917

[148] Persson PB. 2003 was a good year for the american iournal of physiology-regulatory, integrative and comparative physiology. Am J Physiol Regul Integr Comp Physiol. (2004) 286:R607. doi: 10.1152/ajpregu.00015.2004, PMID: 15003940

[149] Hodgetts S Radley H Davies M Grounds MD. Reduced necrosis of dystrophic muscle by depletion of host neutrophils, or blocking TNFalpha function with Etanercept in mdx mice. Neuromuscul Disord. (2006) 16:591–602. doi: 10.1016/j.nmd.2006.06.011, PMID: 16935507

[150] Arecco N Clarke CJ Jones FK Simpson DM Mason D Beynon RJ. Elastase levels and activity are increased in dystrophic muscle and impair myoblast cell survival, proliferation and differentiation. Sci Rep. (2016) 6:24708. doi: 10.1038/srep24708, PMID: 27241590 PMC4886533

[151] Saffarzadeh M Juenemann C Queisser MA Lochnit G Barreto G Galuska SP. Neutrophil extracellular traps directly induce epithelial and endothelial cell death: a predominant role of histones. PloS One. (2012) 7:e32366. doi: 10.1371/journal.pone.0032366, PMID: 22389696 PMC3289648

[152] Wehling-Henricks M Sokolow S Lee JJ Myung KH Villalta SA Tidball JG. Major basic protein-1 promotes fibrosis of dystrophic muscle and attenuates the cellular immune response in muscular dystrophy. Hum Mol Genet. (2008) 17:2280–92. doi: 10.1093/hmg/ddn129, PMID: 18430716 PMC2574717

[153] Juban G Chazaud B. Metabolic regulation of macrophages during tissue repair: insights from skeletal muscle regeneration. FEBS Lett. (2017) 591:3007–21. doi: 10.1002/1873-3468.12703, PMID: 28555751

[154] Oishi Y Manabe I. Macrophages in inflammation, repair and regeneration. Int Immunol. (2018) 30:511–28. doi: 10.1093/intimm/dxy054, PMID: 30165385

[155] Dong J Wu B Tian W. Human adipose tissue-derived small extracellular vesicles promote soft tissue repair through modulating M1-to-M2 polarization of macrophages. Stem Cell Res Ther. (2023) 14:67. doi: 10.1186/s13287-023-03306-7, PMID: 37024970 PMC10080905

[156] Mantovani A Biswas SK Galdiero MR Sica A Locati M. Macrophage plasticity and polarization in tissue repair and remodelling. J Pathol. (2013) 229:176–85. doi: 10.1002/path.4133, PMID: 23096265

[157] Rzosinska K Formanowicz D Formanowicz P. The study of the influence of micro-environmental signals on macrophage differentiation using a quantitative Petri net based model. Arch Control Sci. (2017) 27:331–49. doi: 10.1515/acsc-2017-0022

[158] Chaintreuil P Kerreneur E Bourgoin M Savy C Favreau C Robert G. The generation, activation, and polarization of monocyte-derived macrophages in human Malignancies. Front Immunol. (2023) 14:1178337. doi: 10.3389/fimmu.2023.1178337, PMID: 37143666 PMC10151765

[159] Juhas M Abutaleb N Wang JT Ye J Shaikh Z Sriworarat C. Incorporation of macrophages into engineered skeletal muscle enables enhanced muscle regeneration. Nat BioMed Eng. (2018) 2:942–54. doi: 10.1038/s41551-018-0290-2, PMID: 30581652 PMC6296488

[160] Qi Z Yu Y Su Y Cao B Shao H Yang JJ. M1-type microglia-derived extracellular vesicles overexpressing IL-1R1 promote postoperative cognitive dysfunction by regulating neuronal inflammation. Inflammation. (2023) 46:2254–69. doi: 10.1007/s10753-023-01875-6, PMID: 37505422

[161] Yang G Ni JS Li Y Zha M Tu Y Li K. Acceptor engineering for optimized ROS generation facilitates reprogramming macrophages to M1 phenotype in photodynamic immunotherapy. Angew Chem Int Ed Engl. (2021) 60:5386–93. doi: 10.1002/anie.202013228, PMID: 33236483

[162] Ley K. M1 means kill; M2 means heal. J Immunol. (2017) 199:2191–3. doi: 10.4049/jimmunol.1701135, PMID: 28923980

[163] Yamamoto N Oyaizu T Enomoto M Horie M Yuasa M Okawa A. VEGF and bFGF induction by nitric oxide is associated with hyperbaric oxygen-induced angiogenesis and muscle regeneration. Sci Rep. (2020) 10:2744. doi: 10.1038/s41598-020-59615-x, PMID: 32066777 PMC7026099

[164] Comito G Giannoni E Segura CP Barcellos-de-Souza P Raspollini MR Baroni G. Cancer-associated fibroblasts and M2-polarized macrophages synergize during prostate carcinoma progression. Oncogene. (2014) 33:2423–31. doi: 10.1038/onc.2013.191, PMID: 23728338

[165] Martins L Gallo CC Honda TSB Alves PT Stilhano RS Rosa DS. Skeletal muscle healing by M1-like macrophages produced by transient expression of exogenous GM-CSF. Stem Cell Res Ther. (2020) 11:473. doi: 10.1186/s13287-020-01992-1, PMID: 33158459 PMC7648431

[166] Gaudet AD Popovich PG Ramer MS. Wallerian degeneration: gaining perspective on inflammatory events after peripheral nerve injury. J Neuroinflammation. (2011) 8:110. doi: 10.1186/1742-2094-8-110, PMID: 21878126 PMC3180276

[167] Nikolakopoulou AM Dutta R Chen Z Miller RH Trapp BD. Activated microglia enhance neurogenesis via trypsinogen secretion. Proc Natl Acad Sci U.S.A. (2013) 110:8714–9. doi: 10.1073/pnas.1218856110, PMID: 23650361 PMC3666689

[168] Cheng Z Zhu W Cao K Wu F Li J Wang G. Anti-inflammatory mechanism of neural stem cell transplantation in spinal cord injury. Int J Mol Sci. (2016) 17:1380. doi: 10.3390/ijms17091380, PMID: 27563878 PMC5037660

[169] Hass R von der Ohe J Ungefroren H. Impact of the tumor microenvironment on tumor heterogeneity and consequences for cancer cell plasticity and stemness. Cancers (Basel). (2020) 12:3716. doi: 10.3390/cancers12123716, PMID: 33322354 PMC7764513

[170] Martin KE García AJ. Macrophage phenotypes in tissue repair and the foreign body response: Implications for biomaterial-based regenerative medicine strategies. Acta Biomater. (2021) 133:4–16. doi: 10.1016/j.actbio.2021.03.038, PMID: 33775905 PMC8464623

[171] Hu D Li R Li Y Wang M Wang L Wang S. Inflammation-targeted nanomedicines alleviate oxidative stress and reprogram macrophages polarization for myocardial infarction treatment. Adv Sci (Weinh). (2024) 11:e2308910. doi: 10.1002/advs.202308910, PMID: 38582507 PMC11151042

[172] Hourani T Holden JA Li W Lenzo JC Hadjigol S O'Brien-Simpson NM. Tumor associated macrophages: origin, recruitment, phenotypic diversity, and targeting. Front Oncol. (2021) 11:788365. doi: 10.3389/fonc.2021.788365, PMID: 34988021 PMC8722774

[173] Mantovani A Allavena P Marchesi F Garlanda C. Macrophages as tools and targets in cancer therapy. Nat Rev Drug Discov. (2022) 21:799–820. doi: 10.1038/s41573-022-00520-5, PMID: 35974096 PMC9380983

[174] Patsalos A Halasz L Oleksak D Wei X Nagy G Tzerpos P. Spatiotemporal transcriptomic mapping of regenerative inflammation in skeletal muscle reveals a dynamic multilayered tissue architecture. J Clin Invest. (2024) 134:e173858. doi: 10.1172/jci173858, PMID: 39190487 PMC11473166

[175] Patsalos A Halasz L Medina-Serpas MA Berger WK Daniel B Tzerpos P. A growth factor-expressing macrophage subpopulation orchestrates regenerative inflammation via GDF-15. J Exp Med. (2022) 219:e20210420. doi: 10.1084/jem.20210420, PMID: 34846534 PMC8635277

[176] Zhang X Zhang Y Chen Y Ji Y Lyu Y Miao Z. Unraveling the immune system's role in peripheral nerve regeneration: a pathway to enhanced healing. Front Immunol. (2025) 16:1540199. doi: 10.3389/fimmu.2025.1540199, PMID: 40061948 PMC11885135

[177] Lawrence T. The nuclear factor NF-kappaB pathway in inflammation. Cold Spring Harb Perspect Biol. (2009) 1:a001651. doi: 10.1101/cshperspect.a001651, PMID: 20457564 PMC2882124

[178] Mueller AL Brockmueller A Kunnumakkara AB Shakibaei M. Modulation of inflammation by plant-derived nutraceuticals in tendinitis. Nutrients. (2022) 14:2030. doi: 10.3390/nu14102030, PMID: 35631173 PMC9143056

[179] Jiang L Liu T Lyu K Chen Y Lu J Wang X. Inflammation-related signaling pathways in tendinopathy. Open Life Sci. (2023) 18:20220729. doi: 10.1515/biol-2022-0729, PMID: 37744452 PMC10512452

[180] Xia T Fu S Yang R Yang K Lei W Yang Y. Advances in the study of macrophage polarization in inflammatory immune skin diseases. J Inflammation (Lond). (2023) 20:33. doi: 10.1186/s12950-023-00360-z, PMID: 37828492 PMC10568804

[181] Galozzi P Negm O Bindoli S Tighe P Sfriso P Punzi LA. pro-inflammatory signature constitutively activated in monogenic autoinflammatory diseases. Int J Mol Sci. (2022) 23:1828. doi: 10.3390/ijms23031828, PMID: 35163749 PMC8836675

[182] Zhang C Wang C Li Y Miwa T Liu C Cui W. Complement C3a signaling facilitates skeletal muscle regeneration by regulating monocyte function and trafficking. Nat Commun. (2017) 8:2078. doi: 10.1038/s41467-017-01526-z, PMID: 29233958 PMC5727192

[183] Peng J Han L Liu B Song J Wang Y Wang K. Gli1 marks a sentinel muscle stem cell population for muscle regeneration. Nat Commun. (2023) 14:6993. doi: 10.1038/s41467-023-42837-8, PMID: 37914731 PMC10620419

[184] Wang X Zhou L. The multifaceted role of macrophages in homeostatic and injured skeletal muscle. Front Immunol. (2023) 14:1274816. doi: 10.3389/fimmu.2023.1274816, PMID: 37954602 PMC10634307

[185] Arnold IC Munitz A. Spatial adaptation of eosinophils and their emerging roles in homeostasis, infection and disease. Nat Rev Immunol. (2024) 24:858–77. doi: 10.1038/s41577-024-01048-y, PMID: 38982311

[186] Borrelli C Gurtner A Arnold IC Moor AE. Stress-free single-cell transcriptomic profiling and functional genomics of murine eosinophils. Nat Protoc. (2024) 19:1679–709. doi: 10.1038/s41596-024-00967-3, PMID: 38504138

[187] Gigon L Fettrelet T Yousefi S Simon D Simon HU. Eosinophils from A to Z. Allergy. (2023) 78:1810–46. doi: 10.1111/all.15751, PMID: 37102676

[188] Jackson DJ Pavord ID. Living without eosinophils: evidence from mouse and man. Eur Respir J. (2023) 61:2201217. doi: 10.1183/13993003.01217-2022, PMID: 35953100 PMC9834633

[189] Wechsler ME Munitz A Ackerman SJ Drake MG Jackson DJ Wardlaw AJ. Eosinophils in health and disease: A State-of-the-Art Review. Mayo Clin Proc. (2021) 96:2694–707. doi: 10.1016/j.mayocp.2021.04.025, PMID: 34538424

[190] Takahashi Y Yoda M Tsuji O Horiuchi K Watanabe K Nakamura M. IL-33-ST2 signaling in fibro-adipogenic progenitors alleviates immobilization-induced muscle atrophy in mice. Skelet Muscle. (2024) 14:6. doi: 10.1186/s13395-024-00338-2, PMID: 38561845 PMC10983726

[191] Ji L Zhao X Zhang B Kang L Song W Zhao B. Slc6a8-mediated creatine uptake and accumulation reprogram macrophage polarization via regulating cytokine responses. Immunity. (2019) 51:272–284.e277. doi: 10.1016/j.immuni.2019.06.007, PMID: 31399282

[192] Miyake K Ito J Karasuyama H. Role of basophils in a broad spectrum of disorders. Front Immunol. (2022) 13:902494. doi: 10.3389/fimmu.2022.902494, PMID: 35693800 PMC9186123

[193] Zhao H Wu L Yan G Chen Y Zhou M Wu Y. Inflammation and tumor progression: signaling pathways and targeted intervention. Signal Transduct Target Ther. (2021) 6:263. doi: 10.1038/s41392-021-00658-5, PMID: 34248142 PMC8273155

[194] Wu D Molofsky AB Liang HE Ricardo-Gonzalez RR Jouihan HA Bando JK. Eosinophils sustain adipose alternatively activated macrophages associated with glucose homeostasis. Science. (2011) 332:243–7. doi: 10.1126/science.1201475, PMID: 21436399 PMC3144160

[195] Ruffell D Mourkioti F Gambardella A Kirstetter P Lopez RG Rosenthal N. CREB-C/EBPbeta cascade induces M2 macrophage-specific gene expression and promotes muscle injury repair. Proc Natl Acad Sci U S A. (2009) 106:17475–80. doi: 10.1073/pnas.0908641106, PMID: 19805133 PMC2762675

[196] Raziyeva K Kim Y Zharkinbekov Z Kassymbek K Jimi S Saparov A. Immunology of acute and chronic wound healing. Biomolecules. (2021) 11:700. doi: 10.3390/biom11050700, PMID: 34066746 PMC8150999

[197] Sun L Su Y Jiao A Wang X Zhang BT. cells in health and disease. Signal Transduct Target Ther. (2023) 8:235. doi: 10.1038/s41392-023-01471-y, PMID: 37332039 PMC10277291

[198] Wraith DC. Adaptive T cell tuning in immune regulation and immunotherapy of autoimmune diseases. Immunol Lett. (2022) 244:12–8. doi: 10.1016/j.imlet.2022.02.007, PMID: 35231497

[199] Deyhle MR Carlisle M Sorensen JR Hafen PS Jesperson K Ahmadi M. Accumulation of skeletal muscle T cells and the repeated bout effect in rats. Med Sci Sports Exerc. (2020) 52:1280–93. doi: 10.1249/mss.0000000000002256, PMID: 31876672

[200] Deyhle MR Hyldahl RD. The role of T lymphocytes in skeletal muscle repair from traumatic and contraction-induced injury. Front Physiol. (2018) 9:768. doi: 10.3389/fphys.2018.00768, PMID: 29973887 PMC6019499

[201] Becker M Joseph SS Garcia-Carrizo F Tom RZ Opaleva D Serr I. Regulatory T cells require IL6 receptor alpha signaling to control skeletal muscle function and regeneration. Cell Metab. (2023) 35:1736–1751.e1737. doi: 10.1016/j.cmet.2023.08.010, PMID: 37734370 PMC10563138

[202] Gao Y Yang J Cai Y Fu S Zhang N Fu X. IFN-γ-mediated inhibition of lung cancer correlates with PD-L1 expression and is regulated by PI3K-AKT signaling. Int J Cancer. (2018) 143:931–43. doi: 10.1002/ijc.31357, PMID: 29516506

[203] Xu X Xu J Wu J Hu Y Han Y Gu Y. Phosphorylation-mediated IFN-γR2 membrane translocation is required to activate macrophage innate response. Cell. (2018) 175:1336–1351.e1317. doi: 10.1016/j.cell.2018.09.011, PMID: 30318148

[204] Chen KW Tong YL Yao YM. Advances in the research of effects and regulatory mechanism of regulatory T cells in tissue injury and repair. Zhonghua Shao Shang Za Zhi. (2019) 35:828–32. doi: 10.3760/cma.j.issn.1009-2587.2019.11.013, PMID: 31775475

[205] Huang B Li H Jiang Q Li Y Jiang Z Cao H. Elevated type I IFN signalling directly affects CD8(+) T-cell distribution and autoantigen recognition of the skeletal muscles in active JDM patients. J Autoimmun. (2024) 146:103232. doi: 10.1016/j.jaut.2024.103232, PMID: 38692172

[206] Kamiya M Mizoguchi F Kawahata K Wang D Nishibori M Day J. Targeting necroptosis in muscle fibers ameliorates inflammatory myopathies. Nat Commun. (2022) 13:166. doi: 10.1038/s41467-021-27875-4, PMID: 35013338 PMC8748624

[207] Chalmin F Humblin E Ghiringhelli F Végran F. Transcriptional programs underlying Cd4 T cell differentiation and functions. Int Rev Cell Mol Biol. (2018) 341:1–61. doi: 10.1016/bs.ircmb.2018.07.002, PMID: 30262030

[208] Lam N Lee Y Farber DLA. guide to adaptive immune memory. Nat Rev Immunol. (2024) 24:810–29. doi: 10.1038/s41577-024-01040-6, PMID: 38831162

[209] Wang S Castro BA Katz JL Arrieta V Najem H Vazquez-Cervantes GI. B cell-based therapy produces antibodies that inhibit glioblastoma growth. J Clin Invest. (2024) 134:e177384. doi: 10.1172/jci177384, PMID: 39207859 PMC11473152

[210] Pan Z Li M Zhang P Li T Liu R Liu J. Peripheral blood lymphocyte subsets and heterogeneity of B cell subsets in patients of idiopathic inflammatory myositis with different myositis-specific autoantibodies. Inflammation. (2024) 48:118–32. doi: 10.1007/s10753-024-02052-z, PMID: 38755405

[211] Geladaris A Häusser-Kinzel S Pretzsch R Nissimov N Lehmann-Horn K Häusler D. IL-10-providing B cells govern pro-inflammatory activity of macrophages and microglia in CNS autoimmunity. Acta Neuropathol. (2023) 145:461–77. doi: 10.1007/s00401-023-02552-6, PMID: 36854993 PMC10020302

[212] Laubreton D Drajac C Eléouët JF Rameix-Welti MA Lo-Man R Riffault S. Regulatory B lymphocytes colonize the respiratory tract of neonatal mice and modulate immune responses of alveolar macrophages to RSV infection in IL-10-dependant manner. Viruses. (2020) 12:822. doi: 10.3390/v12080822, PMID: 32751234 PMC7472339

[213] Scala P Rehak L Giudice V Ciaglia E Puca AA Selleri C. Stem cell and macrophage roles in skeletal muscle regenerative medicine. Int J Mol Sci. (2021) 22:10867. doi: 10.3390/ijms221910867, PMID: 34639203 PMC8509639

[214] Alvarez AM DeOcesano-Pereira C Teixeira C Moreira V. IL-1β and TNF-α modulation of proliferated and committed myoblasts: IL-6 and COX-2-derived prostaglandins as key actors in the mechanisms involved. Cells. (2020) 9:2005. doi: 10.3390/cells9092005, PMID: 32882817 PMC7564831

[215] Patel AM Liu YS Davies SP Brown RM Kelly DA Scheel-Toellner D. The role of B cells in adult and paediatric liver injury. Front Immunol. (2021) 12:729143. doi: 10.3389/fimmu.2021.729143, PMID: 34630404 PMC8495195

[216] Gama JFG Romualdo RD de Assis ML de Oliveira LM Quírico-Santos T Alves LA. Role of regulatory T cells in skeletal muscle regeneration: A systematic review. Biomolecules. (2022) 12:817. doi: 10.3390/biom12060817, PMID: 35740942 PMC9220893

[217] Singh MD Ni M Sullivan JM Hamerman JA Campbell DJ. B cell adaptor for PI3-kinase (BCAP) modulates CD8(+) effector and memory T cell differentiation. J Exp Med. (2018) 215:2429–43. doi: 10.1084/jem.20171820, PMID: 30093532 PMC6122975

[218] Karkanitsa M Fathi P Ngo T Sadtler K. Mobilizing endogenous repair through understanding immune reaction with biomaterials. Front Bioeng Biotechnol. (2021) 9:730938. doi: 10.3389/fbioe.2021.730938, PMID: 34917594 PMC8670074

[219] Cui J Zhang YJ Li X Luo JJ Zhao LL Xie XY. Decellularized tendon scaffolds loaded with collagen targeted extracellular vesicles from tendon-derived stem cells facilitate tendon regeneration. J Control Release. (2023) 360:842–57. doi: 10.1016/j.jconrel.2023.07.032, PMID: 37478916

[220] Walczak PA Perez-Esteban P Bassett DC Hill EJ. Modelling the central nervous system: tissue engineering of the cellular microenvironment. Emerg Top Life Sci. (2021) 5:507–17. doi: 10.1042/etls20210245, PMID: 34524411 PMC8589431

[221] No YJ Castilho M Ramaswamy Y Zreiqat H. Role of biomaterials and controlled architecture on tendon/ligament repair and regeneration. Adv Mater. (2020) 32:e1904511. doi: 10.1002/adma.201904511, PMID: 31814177

[222] Beldjilali-Labro M Garcia Garcia A Farhat F Bedoui F Grosset JF Dufresne M. Biomaterials in tendon and skeletal muscle tissue engineering: Current trends and challenges. Materials (Basel). (2018) 11:1116. doi: 10.3390/ma11071116, PMID: 29966303 PMC6073924

[223] Zhao W Hu C Xu T. *In vivo* bioprinting: Broadening the therapeutic horizon for tissue injuries. Bioact Mater. (2023) 25:201–22. doi: 10.1016/j.bioactmat.2023.01.018, PMID: 36817820 PMC9932583

[224] Han S Cruz SH Park S Shin SR. Nano-biomaterials and advanced fabrication techniques for engineering skeletal muscle tissue constructs in regenerative medicine. Nano Converg. (2023) 10:48. doi: 10.1186/s40580-023-00398-y, PMID: 37864632 PMC10590364

[225] Tang Y Wang Z Xiang L Zhao Z Cui W. Functional biomaterials for tendon/ligament repair and regeneration. Regener Biomater. (2022) 9:rbac062. doi: 10.1093/rb/rbac062, PMID: 36176715 PMC9514853

[226] Schneider C. Traumeel - an emerging option to nonsteroidal anti-inflammatory drugs in the management of acute musculoskeletal injuries. Int J Gen Med. (2011) 4:225–34. doi: 10.2147/ijgm.S16709, PMID: 21556350 PMC3085232

[227] Chan KM Fu SC. Anti-inflammatory management for tendon injuries - friends or foes? Sports Med Arthrosc Rehabil Ther Technol. (2009) 1:23. doi: 10.1186/1758-2555-1-23, PMID: 19825161 PMC2770552

[228] Sass FA Fuchs M Pumberger M Geissler S Duda GN Perka C. Immunology guides skeletal muscle regeneration. Int J Mol Sci. (2018) 19:835. doi: 10.3390/ijms19030835, PMID: 29534011 PMC5877696

[229] Kim HJ Kim YH. Comprehensive insights into keloid pathogenesis and advanced therapeutic strategies. Int J Mol Sci. (2024) 25:. doi: 10.3390/ijms25168776, PMID: 39201463 PMC11354446

[230] van Eeden SF Akata K. Macrophages-the immune effector guardians of the lung: impact of corticosteroids on their functional responses. Clin Sci (Lond). (2020) 134:1631–5. doi: 10.1042/cs20200382, PMID: 32608490 PMC7330501

[231] Gust J Ponce R Liles WC Garden GA Turtle CJ. Cytokines in CAR T cell-associated neurotoxicity. Front Immunol. (2020) 11:577027. doi: 10.3389/fimmu.2020.577027, PMID: 33391257 PMC7772425

[232] Li J Tan J Martino MM Lui KO. Regulatory T-cells: potential regulator of tissue repair and regeneration. Front Immunol. (2018) 9:585. doi: 10.3389/fimmu.2018.00585, PMID: 29662491 PMC5890151

[233] Hanna BS Yaghi OK Langston PK Mathis D. The potential for Treg-enhancing therapies in tissue, in particular skeletal muscle, regeneration. Clin Exp Immunol. (2023) 211:138–48. doi: 10.1093/cei/uxac076, PMID: 35972909 PMC10019136

[234] Lei H Schmidt-Bleek K Dienelt A Reinke P Volk HD. Regulatory T cell-mediated anti-inflammatory effects promote successful tissue repair in both indirect and direct manners. Front Pharmacol. (2015) 6:184. doi: 10.3389/fphar.2015.00184, PMID: 26388774 PMC4557110

[235] Alshoubaki YK Nayer B Das S Martino MM. Modulation of the activity of stem and progenitor cells by immune cells. Stem Cells Transl Med. (2022) 11:248–58. doi: 10.1093/stcltm/szab022, PMID: 35303109 PMC8968657

[236] Wang C Liu Y Wang Y Wei Z Suo D Ning G. Low−frequency pulsed electromagnetic field promotes functional recovery, reduces inflammation and oxidative stress, and enhances HSP70 expression following spinal cord injury. Mol Med Rep. (2019) 19:1687–93. doi: 10.3892/mmr.2019.9820, PMID: 30628673 PMC6390012

[237] Mata R Yao Y Cao W Ding J Zhou T Zhai Z. The dynamic inflammatory tissue microenvironment: Signality and disease therapy by biomaterials. Res (Wash D C). (2021) 2021:4189516. doi: 10.34133/2021/4189516, PMID: 33623917 PMC7879376

[238] Yousefpour P Ni K Irvine DJ. Targeted modulation of immune cells and tissues using engineered biomaterials. Nat Rev Bioeng. (2023) 1:107–24. doi: 10.1038/s44222-022-00016-2, PMID: 37772035 PMC10538251

[239] Ou Q Power R Griffin MD. Revisiting regulatory T cells as modulators of innate immune response and inflammatory diseases. Front Immunol. (2023) 14:1287465. doi: 10.3389/fimmu.2023.1287465, PMID: 37928540 PMC10623442

[240] Rosado MM Simkó M Mattsson MO Pioli C. Immune-modulating perspectives for low frequency electromagnetic fields in innate immunity. Front Public Health. (2018) 6:85. doi: 10.3389/fpubh.2018.00085, PMID: 29632855 PMC5879099

[241] Mashayekhi K Khazaie K Faubion WAJR Kim GB. Biomaterial-enhanced treg cell immunotherapy: A promising approach for transplant medicine and autoimmune disease treatment. Bioact Mater. (2024) 37:269–98. doi: 10.1016/j.bioactmat.2024.03.030, PMID: 38694761 PMC11061617

[242] Wu F Wang P Wei X Yang Y Al Mamun A Zhang X. Barrier-penetrating liposome targeted delivery of basic fibroblast growth factor for spinal cord injury repair. Mater Today Bio. (2023) 18:100546. doi: 10.1016/j.mtbio.2023.100546, PMID: 36691606 PMC9860515

[243] Stuart T Butler A Hoffman P Hafemeister C Papalexi E Mauck WM 3rd. Comprehensive integration of single-cell data. Cell. (2019) 177:1888–1902.e1821. doi: 10.1016/j.cell.2019.05.031, PMID: 31178118 PMC6687398

[244] Liu W Puri A Fu D Chen L Wang C Kellis M. Dissecting the tumor microenvironment in response to immune checkpoint inhibitors via single-cell and spatial transcriptomics. Clin Exp Metastasis. (2024) 41:313–32. doi: 10.1007/s10585-023-10246-2, PMID: 38064127 PMC11374862

[245] Tirosh I Izar B Prakadan SM Wadsworth MH 2nd Treacy D Trombetta JJ. Dissecting the multicellular ecosystem of metastatic melanoma by single-cell RNA-seq. Science. (2016) 352:189–96. doi: 10.1126/science.aad0501, PMID: 27124452 PMC4944528

[246] Weavers H Martin P. The cell biology of inflammation: From common traits to remarkable immunological adaptations. J Cell Biol. (2020) 219:e202004003. doi: 10.1083/jcb.202004003, PMID: 32539109 PMC7337495

[247] Ståhl PL Salmén F Vickovic S Lundmark A Navarro JF Magnusson J. Visualization and analysis of gene expression in tissue sections by spatial transcriptomics. Science. (2016) 353:78–82. doi: 10.1126/science.aaf2403, PMID: 27365449

[248] Davis-Marcisak EF Deshpande A Stein-O'Brien GL Ho WJ Laheru D Jaffee EM. From bench to bedside: Single-cell analysis for cancer immunotherapy. Cancer Cell. (2021) 39:1062–80. doi: 10.1016/j.ccell.2021.07.004, PMID: 34329587 PMC8406623

[249] Peterson EA Sun J Wang J. Leukocyte-mediated cardiac repair after myocardial infarction in non-regenerative vs. regenerative systems. J Cardiovasc Dev Dis. (2022) 9:63. doi: 10.3390/jcdd9020063, PMID: 35200716 PMC8877434

[250] Dobie R Henderson NC. Unravelling fibrosis using single-cell transcriptomics. Curr Opin Pharmacol. (2019) 49:71–5. doi: 10.1016/j.coph.2019.09.004, PMID: 31670054

[251] Chen W Li C Chen Y Bin J Chen Y. Cardiac cellular diversity and functionality in cardiac repair by single-cell transcriptomics. Front Cardiovasc Med. (2023) 10:1237208. doi: 10.3389/fcvm.2023.1237208, PMID: 37920179 PMC10619858

[252] Zhang B Su Y Zhou J Zheng Y Zhu D. Toward a better regeneration through implant-mediated immunomodulation: harnessing the immune responses. Adv Sci (Weinh). (2021) 8:e2100446. doi: 10.1002/advs.202100446, PMID: 34117732 PMC8373114

[253] Shanley LC Mahon OR Kelly DJ Dunne A. Harnessing the innate and adaptive immune system for tissue repair and regeneration: Considering more than macrophages. Acta Biomater. (2021) 133:208–21. doi: 10.1016/j.actbio.2021.02.023, PMID: 33657453

[254] Ferreira LMR Muller YD Bluestone JA Tang Q. Next-generation regulatory T cell therapy. Nat Rev Drug Discov. (2019) 18:749–69. doi: 10.1038/s41573-019-0041-4, PMID: 31541224 PMC7773144

[255] Yang SJ Singh AK Drow T Tappen T Honaker Y Barahmand-Pour-Whitman F. Pancreatic islet-specific engineered T(regs) exhibit robust antigen-specific and bystander immune suppression in type 1 diabetes models. Sci Transl Med. (2022) 14:eabn1716. doi: 10.1126/scitranslmed.abn1716, PMID: 36197963

[256] Liu Y Segura T. Biomaterials-mediated regulation of macrophage cell fate. Front Bioeng Biotechnol. (2020) 8:609297. doi: 10.3389/fbioe.2020.609297, PMID: 33363135 PMC7759630

[257] Li J Jiang X Li H Gelinsky M Gu Z. Tailoring materials for modulation of macrophage fate. Adv Mater. (2021) 33:e2004172. doi: 10.1002/adma.202004172, PMID: 33565154 PMC9245340

[258] Zarubova J Hasani-Sadrabadi MM Ardehali R Li S. Immunoengineering strategies to enhance vascularization and tissue regeneration. Adv Drug Delivery Rev. (2022) 184:114233. doi: 10.1016/j.addr.2022.114233, PMID: 35304171 PMC10726003

[259] MacDonald KG Hoeppli RE Huang Q Gillies J Luciani DS Orban PC. Alloantigen-specific regulatory T cells generated with a chimeric antigen receptor. J Clin Invest. (2016) 126:1413–24. doi: 10.1172/jci82771, PMID: 26999600 PMC4811124

[260] Kwee BJ Mooney DJ. Biomaterials for skeletal muscle tissue engineering. Curr Opin Biotechnol. (2017) 47:16–22. doi: 10.1016/j.copbio.2017.05.003, PMID: 28575733 PMC5617779

